# Climate-driven variation in the phenology of juvenile *Ixodes pacificus* on lizard hosts

**DOI:** 10.1186/s13071-025-06749-4

**Published:** 2025-04-15

**Authors:** Samantha Sambado, Amanda Sparkman, Andrea Swei, Andrew J. MacDonald, Hillary S. Young, Jordan Salomon, Arielle Crews, Kacie Ring, Stephanie Copeland, Cheryl J. Briggs

**Affiliations:** 1 Ecology, Evolution and Marine Biology Department, University of California Santa Barbara, Santa Barbara, CA USA; 2 Biology Department, Westmont College, Santa Barbara, CA USA; 3 Biology Department, San Francisco State University, San Francisco, CA USA; 4 Bren School of Environmental Science and Management, University of California Santa Barbara, Santa Barbara, CA USA; 5 Ecology and Evolutionary Biology Program, Texas A&M University, College Station, TX USA; 6 San Mateo County Mosquito and Vector Control District, Burlingame, CA USA

**Keywords:** *Ixodes pacificus*, *Sceloporus occidentalis*, Latitudinal gradient, Mediterranean climate, Host-attached ticks, Phenology

## Abstract

**Background:**

Ectothermic arthropods, like ticks, are sensitive indicators of environmental changes, and their seasonality plays a critical role in the dynamics of tick-borne disease in a warming world. Juvenile tick phenology, which influences pathogen transmission, may vary across climates, with longer tick seasons in cooler climates potentially amplifying transmission. However, assessing juvenile tick phenology is challenging in arid climates because ticks spend less time seeking for blood meals (i.e. questing) due to desiccation pressures. As a result, traditional collection methods like dragging or flagging are less effective. To improve our understanding of juvenile tick seasonality across a latitudinal gradient, we examined *Ixodes pacificus* phenology on lizards, the primary juvenile tick host in California, and explored how climate factors influence phenological patterns.

**Methods:**

Between 2013 and 2022, ticks were removed from 1527 lizards at 45 locations during peak tick season (March–June). Tick counts were categorized by life stage (larvae and nymphs) and linked with remotely sensed climate data, including monthly maximum temperature, specific humidity and Palmer Drought Severity Index (PDSI). Juvenile phenology metrics, including tick abundances on lizards, Julian date of peak mean abundance and temporal overlap between larval and nymphal populations, were analyzed along a latitudinal gradient. Generalized additive models (GAMs) were applied to assess climate-associated variation in juvenile abundance on lizards.

**Results:**

Mean tick abundance per lizard ranged from 0.17 to 47.21 across locations, with the highest abundance in the San Francisco Bay Area and lowest in Los Angeles, where more lizards had zero ticks attached. In the San Francisco Bay Area, peak nymphal abundance occurred 25 days earlier than peak larval abundance. Temporal overlap between larval and nymphal stages at a given location varied regionally, with northern areas showing higher overlap, possibly due to the bimodal seasonality of nymphs. We found that locations with higher temperatures and increased drought stress were linked to lower tick abundances, although the magnitude of these effects depended on regional location.

**Conclusions:**

Our study, which compiled 10 years of data, reveals significant regional variation in juvenile *I. pacificus* phenology across California, including differences in abundance, peak timing, and temporal overlap. These findings highlight the influence of local climate on tick seasonality, with implications for tick-borne disease dynamics in a changing climate.

**Graphical Abstract:**

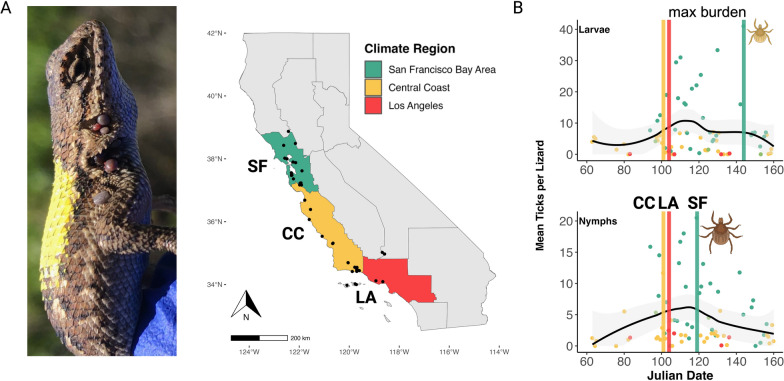

**Supplementary Information:**

The online version contains supplementary material available at 10.1186/s13071-025-06749-4.

## Background

Pathogen vectors, such as ticks, pose serious risks to the health of both humans and animals [1]. Ticks, as ectotherms, are highly sensitive to environmental conditions, and their population dynamics—including abundance, seasonality and host associations—are significantly affected by weather and climate conditions [2–4]. Notably, juvenile ticks, especially larval and nymphal *Ixodes* species, exhibit seasonal activity patterns that directly impact local pathogen prevalence of non-vertically transmitted pathogens such as *Borrelia burgdorferi*, the causative agent of Lyme disease [5–7]. For example, if uninfected larvae emerge prior to infected nymphs, the opportunities for acquiring infectious blood meals diminish [7]; conversely, the emergence of infected nymphs prior to larvae can enhance pathogen transmission by increasing the likelihood of larvae feeding on an infected blood meal and becoming infectious nymphs in subsequent seasons [7].

Lyme disease is a key example of how the seasonality of juvenile ticks can influence the risk to human health. The prevalence of Lyme disease is highly variable across the USA, even in areas where environmental conditions favor both competent *Ixodes* ticks and their vertebrate hosts [8–10]. Various ecological hypotheses have been proposed to explain these discrepancies, including differences in vertebrate community structures, habitat fragmentation that increases human-tick interactions and strain-specific variations in the Lyme disease bacterium [11–13]. However, many studies focus on small spatial scales when examining the relationship between these factors and disease risk, potentially overlooking the critical influence of larger scale climatic factors across the full range of *Ixodes scapularis* and *Ixodes pacificus*.

Linking climatic conditions to juvenile *Ixodes* seasonality is essential for explaining geographic patterns of Lyme disease risk and implications for how climate change may alter this risk. For example, juvenile seasonality has been cited as an explanation for heightened Lyme disease incidence in the cooler northeastern USA compared to the warmer southeastern regions [3, 14–17]. However, substantial time and resource constraints often hinder systematic longitudinal and latitudinal studies [16, 18]. Latitudinal studies that have successfully collected juvenile ticks in drier regions have typically relied on sampling juvenile tick hosts, such as lizards, rather than using traditional tick collection methods, such as dragging or flagging [14, 19, 20]. Sampling juvenile ticks in drier regions presents challenges due to lower overall tick abundances, fragmented distributions and the behavioral adaptations of ticks to desiccation pressures. These ticks are more likely to seek ground-level leaf litter where desiccation risk is reduced and hosts like lizards and rodents are more common rather than questing in aboveground vegetation [21–24]. Even in regions with less intense desiccation, sentinel hosts like deer and lizards can effectively detect ticks, even when regional tick densities are low [19, 25, 26]. Lizards are the preferred host for juvenile *I. pacificus* and, consequently, lizards typically have higher tick burdens than rodents captured within the same spatial and temporal context [27–29]. Although lizards do not directly provide infectious blood meals, lizards can serve as reliable proxies for broader juvenile tick activity patterns, offering insights into tick burden dynamics on rodents.

California’s western coastal region is particularly well-suited for investigating climate effects on juvenile phenology patterns for several reasons. First, Lyme disease risk is disproportionately high in northwestern California, with Mendocino County reporting an incidence rate of 3.9 cases per 100,000 people, compared to the state average of 0.2 [30] (see California Department of Public Health [CDPH] 2021 https://www.cdph.ca.gov/Programs/CID/DCDC/CDPH%20Document%20Library/LymeDiseaseEpiSummary2013-2019.pdf). Second, *I. pacificus* ticks and their preferred larval/nymphal host, the western fence lizard (*Sceloporus occidentalis*), share a broad latitudinal distribution [10]. Third, the region encompasses significant microclimate variability, with coastal areas exhibiting higher humidity levels and lower temperatures than nearby inland areas [19, 31, 32]. Finally, areas with significant desiccation stress have historically shown limited success in assessing juvenile tick activity using traditional sampling methods such as flagging or dragging [20, 22, 33]. Despite these factors, a systematic statewide analysis focusing on the preferred host of juvenile *I. pacificus* has not been conducted.

The aim of this study was to investigate juvenile phenology patterns across California’s latitudinal gradient in relation to climate variability, addressing key gaps in juvenile tick ecology and providing insight into potential ecological mechanisms driving variations in Lyme disease risk. We address the challenges encountered in collecting juvenile ticks in drier regions by incorporating data from lizard surveys and documenting attached juvenile *I. pacificus* across a latitudinal gradient, thereby creating a unique comprehensive dataset. By integrating existing tick research with high-resolution climate data and using generalized additive models (GAMs) [34], we have two main objectives: (i) to characterize seasonal patterns of juvenile tick abundance on their preferred host across latitudes; and (ii) to explore the relationship between juvenile abundances and climate conditions across California’s climatically diverse regions. Understanding the seasonality of juvenile ticks and their climate-driven variation will enhance Lyme disease risk predictions and inform public health strategies, as well as guide future empirical tick-related research.

## Methods

The data for this study were collected from 2013 to 2022, primarily during the peak juvenile activity months of *I. pacificus* ticks, which are March through June. However, most of the northern sites were not sampled until April due to the on average cooler weather. This aggregated dataset includes 45 unique sampling locations and encompasses a total of 253 sampling days (Additional file 1: Table S1; Table S2). Of the 45 locations, 93% (*n* = 42) were sampled multiple times, with 84% (*n* = 38) of locations being sampled ≥ 3 times (Additional file 2: Figure S1). The data reflect the collective efforts of various laboratory groups engaged in ecological research, which included both lizard sampling and tick burden assessments (Additional file 1: Table S1; Additional file 2: Figure S2). The collectors (authors SS, AJM, AmS, AnS, HSY) led the original study design [19, 20, 35, 36] (Table 1).

**Table 1:** Juvenile phenology metrics for three climate regions of California collected from 2013 to 2022

Climate regions	Abundance^a^		Overlap metrics^b^		Julian day of peak abundances^c^	
	Larvae	Nymphs	KDE	Jaccard index	Larvae	Nymphs
San Francisco	12.56 ± 19.1	9.39 ± 10.7	0.89	0.47	144	119
Central Coast	3.32 ± 5.5	2.56 ± 3.9	0.32	0.38	101	101
Los Angeles	0.32 ± 0.9	1.25 ± 1.7	0.30	0.22	104	104

^a^Density measures are presented as the mean ± standard deviations of tick abundances per region

^b^Overlap metrics (kernel density estimation [KDE] and Jaccard index) assess the degree of synchrony between larval and nymphal populations, with higher values indicating more overlap or similarity

^c^Peak abundance refers to the Julian date when the mean abundance was highest in each region. For reference, Julian dates 101 and 144 correspond to April 11th and May 24th, respectively

### Study area

**Fig. 1:**
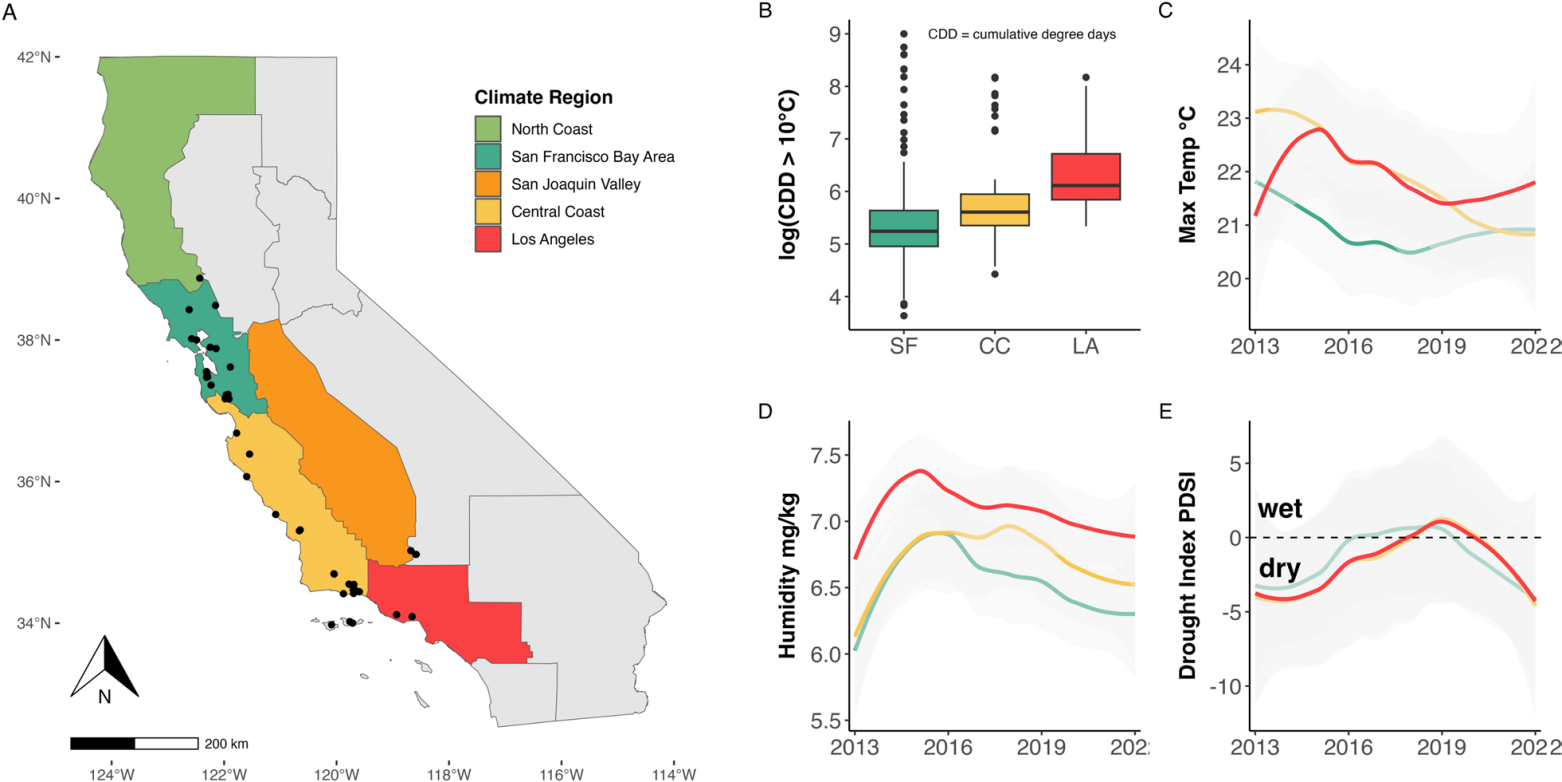
**A** Map of California, color-coded by climate regions. Black dots indicate unique sampling locations where lizard-attached ticks were collected. **B** Box plot showing log_10_ transformed CDD across the most frequently sampled climate regions. **C**–**E** Mean monthly maximum temperature (**C**), mean monthly specific humidity (**D**) and mean drought index (PDSI); shaded gray areas represent 95% confidence intervals. CC, Central Coast; CDD, cumulative degree days; LA, Los Angeles; PDSI, Palmer Drought Severity Index; SF, San Francisco Bay Area

Sampling was conducted across various locations in California, primarily in the western coastal region. This region has a Mediterranean climate, characterized by relatively wet, cool winters that transition into warm, dry summers [37]. Sampling locations spanned a latitudinal gradient from Lake County in the north (39°05′24.00″ N, 122°45′36.00″ W) to Los Angeles County in the south (34°16′50.96″ N, 119°17′40.56″ W) (Fig. 1). These sites were situated in ecosystems known to harbor tick populations, typically including mixed oak woodlands, oak savannas or coastal chaparral habitats [10, 33]. Additional details about the study locations can be found in Additional file 2: Figure S1; Figure S2; Table S1) and in previously published studies [19, 20, 31, 36]. To establish climatically relevant groupings of field sites across our study area, we overlaid location coordinates onto Cal-Adapt’s climate region polygons (North Coast Region, San Francisco Bay Area Region, San Joaquin Valley Region, Central Coast Region, Los Angeles Region), as defined by California’s 4th Climate Change Assessment [38]. A large portion of sampling locations in the Los Angeles Region came from Santa Cruz Island and Santa Rosa Island.

### Field collection

Lizards were primarily captured using standard lassoing techniques, which involved a dental floss or fishing line lasso attached to the tip of a fishing pole, following the methodology of Swei et al. [39]. In some instances, lizards were captured by hand from under cover boards. Sampling typically occurred midday to target peak basking times. Ticks found on a lizard’s ears and nuchal pouches were carefully removed with fine-tipped forceps and placed in 70% ethanol for later identification in the laboratory. Detailed methods for each collector can be found in Additional file 3: Text S1.

### Remotely sensed climate data

All climate data in this study were obtained from the gridded meteorological dataset, gridMET [40]. This dataset provides daily, high-resolution (approx. 4 km) meteorological data across the contiguous USA. In line with the tick-climate literature [41–43], the primary climate variables used in our analysis included near-surface specific humidity (sph; kg/kg), maximum temperature (tmmx; °C) and minimum temperature (tmmn; °C). Derived variables included the monthly Palmer Drought Severity Index (PDSI), a standardized index that estimates relative soil moisture conditions based on a simplified soil water balance, calculated every 5 days. For example, a PDSI value > 4 indicates very wet conditions, while a value < - 4 signifies extreme drought. For each site location coordinates (*n* = 45), data were downloaded for the continuous period from 2013 to 2022 to calculate various indices, such as mean maximum and minimum temperature in each season (spring, summer, fall, winter), and mean climate variables for all months. Using maximum and minimum temperatures, we calculated the cumulative degree days (CDD) for each year and location from 1 January to 31 March. This date range was selected to capture spring dynamics before the peak juvenile season, based on the assumption that a warmer spring (i.e. higher CDD) would result in earlier tick activity [44]. The base temperature was set at 10 °C, a known threshold for the thermal accumulation required by ectothermic invertebrates in California [45]. Daily climate variables were matched to the date of lizard-tick sampling. The gridMET data were accessed on 23 October 2024.

### Statistical analyses

In our analysis, we focus on the abundance of ticks collected from individual lizards, which we aggregate to represent the mean tick abundance for each location or climate region per month. We further categorize the tick abundances by life stages—specifically larval and nymphal ticks. Since most sampling locations (*n* = 41) are concentrated in three regions, we focus our analysis on the San Francisco Bay Area, Central Coast and Los Angeles regions. However, in the Additional files we include summary statistics for two less frequently sampled but important areas of comparison: the North Coast and San Joaquin Valley regions (Additional file 1:  Table S1; Additional file 2: Figure S1; Additional file 3: Text S2; Figure S1).

#### Characterizing the seasonal patterns of juvenile ticks

Juvenile seasonal patterns are characterized by several metrics that capture various aspects of tick population dynamics, including phenology [46, 47]. We constrained our analysis to tick abundance data collected between 1 March and 30 June, which coincides with peak juvenile tick activity and corresponds to Julian dates 60 to 181 in non-leap years. During this period, we quantified the following juvenile seasonal patterns for each climate region and life stage: juvenile abundances on lizards, key phenological dates (e.g. date of peak abundance) and temporal overlap metrics. More details on these methods are found in the following text.

Juvenile abundance refers to the number of ticks removed from individual lizards, with the mean and standard deviation (SD) calculated per month and within each sampling location. Key phenological dates include the Julian date of peak abundance (i.e. the day of maximum mean abundance for each year) and the first and last appearance dates for each life stage. However, due to differences in sampling schedules, the first and last appearance dates serve as general indicators rather than definitive markers for a given region. Finally, we assess temporal overlap between larval and nymphal populations at each location using two metrics: kernel density estimation (KDE) and the Jaccard similarity coefficient (Jaccard index [JI]). KDE is a non-parametric method used to estimate population density, providing a smooth estimate of overlap between larval and nymphal distributions. The JI evaluates the degree of temporal overlap between the two life stages at each location. Together, these metrics provide insights into the temporal dynamics of juvenile tick populations. Higher values for both KDE and JI indicate greater overlap between larval and nymphal populations, implying more synchronized activity within a location. For further justification of these methods, see Additional file 3: Text S2.

#### Exploring the relationship between juvenile tick abundances and climate

We hypothesize that, as ectotherms, tick burdens are influenced by abiotic factors, particularly monthly temperatures, that trigger seasonal behaviors and sufficient humidity to mitigate daily desiccation pressures. Additionally, we aim to capture the long-term effects of local drought conditions on tick populations, if any. We fit GAMs to assess the relationship between tick burdens (i.e. tick count per lizard, including lizards with no ticks) and monthly abiotic variables of the sampling month, including tmmx, sph and the drought index (PDSI) [34]. These variables were standardized for interpretation and checked for multicollinearity (variance inflation factor [vif] < 3).

To capture potential nonlinear relationships between tick abundance and climate variables, we used cubic regression spline smoothing terms (bs = “cr”) for continuous predictors and included an interaction term between climate region and predictors to account for geographic variation in climate and tick burdens. To address spatial autocorrelation, we include Gaussian processing smoothing terms (bs = “gp”) for the latitude and longitude of each sampling location. The optimal smoothing parameter for each smooth term was determined using restricted maximum likelihood (REML) estimation [34]. The model was specified using a Tweedie distribution and a log link function, which is appropriate for modeling overdispersed, zero-inflated count data like tick burdens [48]. Due to limited observations in the North Coast and San Joaquin Valley regions and their relatively close geographic distances from other climate regions, we grouped those locations with the San Francisco Bay Area and Los Angeles regions, respectively. We ran this model first for juvenile tick abundances from individual lizards, followed by separate models for larval and nymphal burdens.

To evaluate model performance, we used the `mgcv` package to fit both GAMs and perform model diagnostics [34]. Within each model grouping, we compared fit using Akaike’s Information Criterion (AIC), selecting the model with the lowest AIC as the best-fitting model. The significance of each predictor was evaluated using likelihood ratio tests based on the estimated degrees of freedom (*edf*), and the model fit was compared to a null model with no smooth terms. We visualized the relationship of our outcomes to predictive values using the plot function to produce partial effects plots that show the predictor’s effect when other variables are at their average value. Additional model justifications are provided in Additional file 3: Text S2 and Additional file 2: Figure S1.

### Software

All statistical analyses were conducted in RStudio version 4.4.1 [49] with a significance level set at *P* < 0.05. Climate data from gridMET were obtained through the ‘climateR` package [50]. Data cleaning and visualizations were conducted with ‘tidyverse’ and ‘ggplot2’ packages, respectively [51, 52]. To assess multicollinearity among climate variables, we used the vif function from the ‘car’ package [53]. The code for figures and analysis is available at: https://github.com/sbsambado/ca_lizardburden.

## Results

### Field collection summary

In total, 1527 individual lizards were sampled. The majority (62%) of the identified lizards were western fence lizards (*Sceloporus occidentalis*), while the remainder included alligator lizards (*Elgaria* spp; 37%) and common side-blotched lizards (*Uta stansburiana*; < 1%). Unidentified lizards were presumed to be *S. occidentalis* based on the objectives of each collecting group. Consequently, we assumed that the majority of attached ticks processed were *I. pacificus*, supported by historical data on tick attachment to *S. occidentalis*, *Elgaria* spp. and *U. stansburiana* [27, 28, 54]. Throughout the study period, a total of 9338 ticks were counted on lizards. However, not all ticks were identified to larval or nymphal stages due to damage sustained during the removal process from lizards. Among those identified, 4197 were larvae and 3239 were nymphs, representing 80% of the attached juvenile ticks. Of the ticks identified to life stage, 100% of ticks were *I. pacificus*.

### Seasonal patterns of juvenile ticks vary

#### Tick abundances

**Fig. 2:**
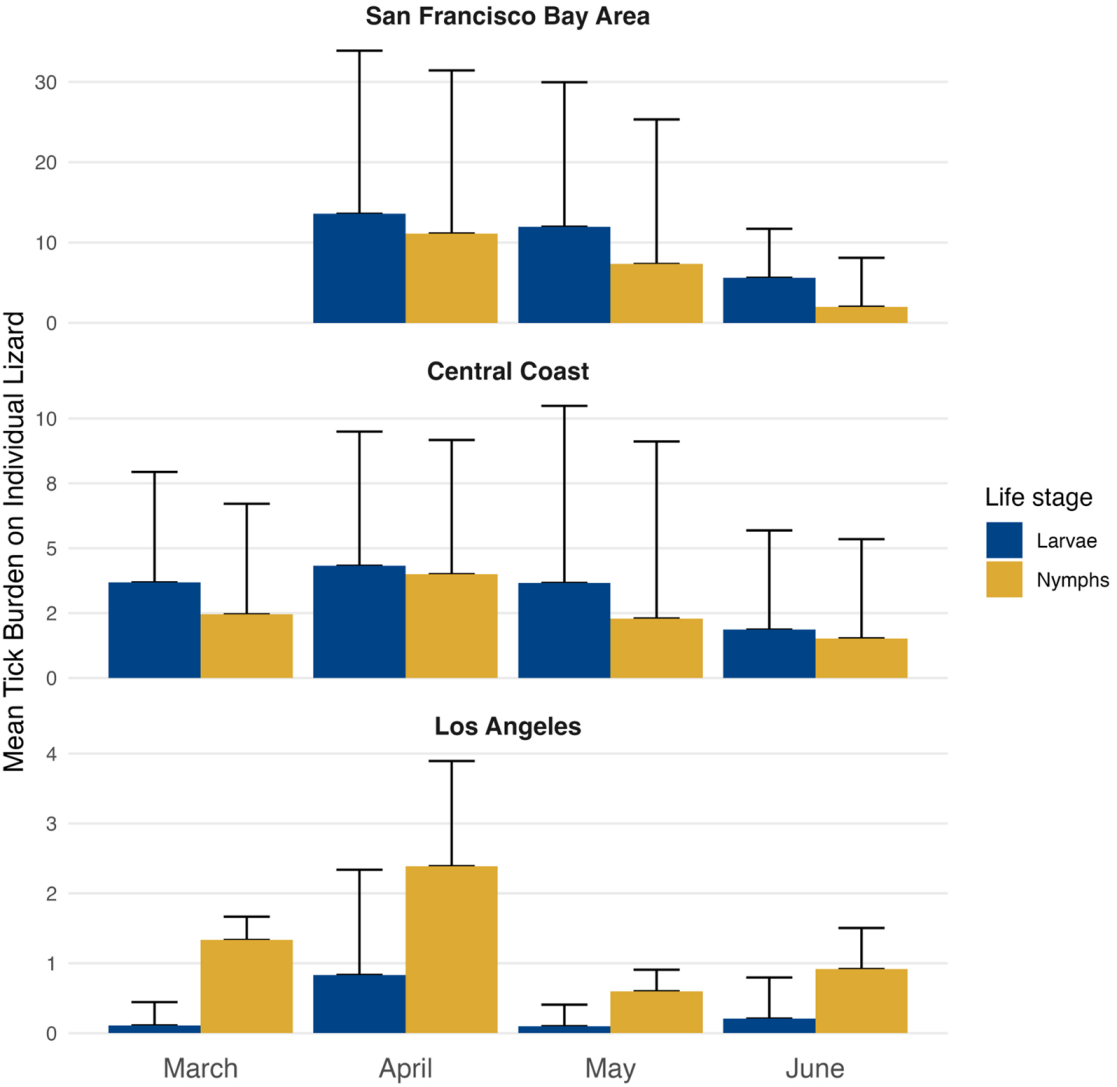
Mean tick abundances across lizards per month, broken down by life stage and climate region. Upper error bars represent the standard error of the mean

 Tick abundances on lizards exhibited considerable variability across our sampling locations, revealing several notable trends. The mean tick abundances on lizards ranged from 0.17 to 47.21 across locations and latitudinally declined, with the San Francisco Bay Area exhibiting the highest mean (± SD) tick burden (18.40 ± 23.07), followed by the Central Coast (3.84 ± 6.62) and Los Angeles region (1.64 ± 3.99). Key regional patterns included: (i) San Francisco Bay Area had the highest mean tick burden across all locations (18 ticks per lizard); (ii) the Los Angeles region had the greatest proportion of lizards with zero ticks (> 50% of sampled lizards); and (iii) peak mean nymphal densities occurred 25 days earlier than peak larval densities in the San Francisco Bay Area only (Fig. 2). Additional results for juvenile ticks by climate region can be found in Additional file 4: Figure S1; Additional file 4: Figure S2.

#### Phenological dates

 The Julian date of peak abundance—when the maximum mean burden per location was reached—for both larvae and nymphs on lizards was similar in the Central Coast and Los Angeles regions (Julian date approx. 100 = April 9th). In contrast, the San Francisco Bay Area experienced peak abundance later in the season. Notably, nymphs in the San Francisco Bay Area reached their peak abundance earlier (Julian date 119 = April 29th) than larvae, whose peak abundance occurred later (Julian date 144 = May 24). Other notable phenological dates include the first Julian dates of appearance, which varied by region (Additional file 4: Table S1). The earliest tick appearance occurred in the Central Coast (Julian date 63 = March 4), followed by Los Angeles region (Julian date 83 = March 24) and then by the San Francisco Bay Area (Julian date 94 = April 4). It should be noted that majority of San Francisco Bay Area sites were not sampled until April, although attempts to capture lizards in the region were made in March, yet no lizards were visually spotted.

#### Overlap metrics

 Both KDE and the JI revealed regional variation in the seasonal overlap between larval and nymphal populations on lizards. The San Francisco Bay Area exhibited the highest overlap (KDE = 0.89, JI = 0.47), followed by the Central Coast (KDE = 0.32, JI = 0.38) and the Los Angeles region (KDE = 0.30, JI = 0.22) (Additional file 4: Table S1).

### Climate-associated patterns of juvenile tick abundances

**Fig. 3:**
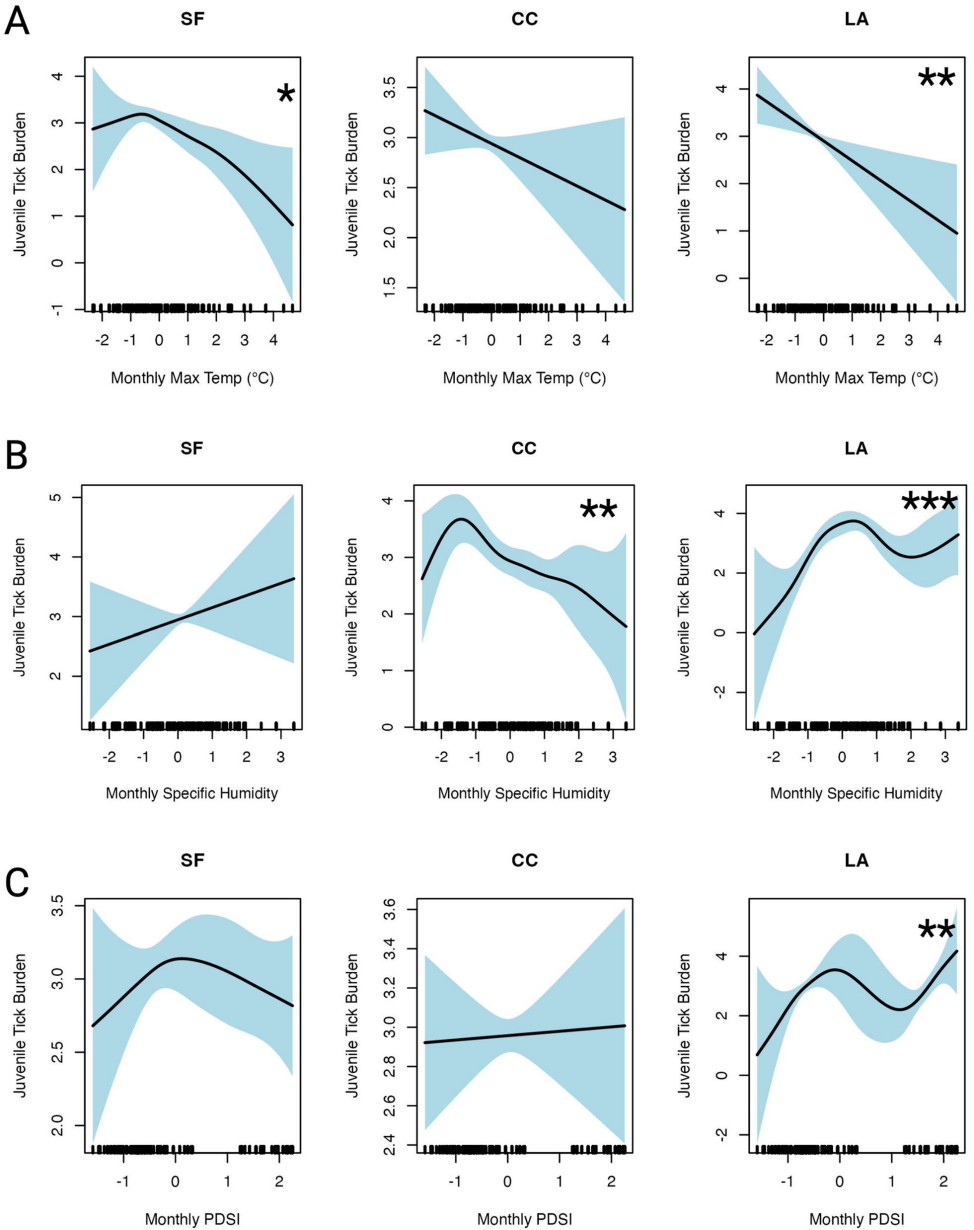
Smooth effect of climate predictors on total tick abundances across California from 2013 to 2022 from the generalized additive model, stratified by climate region. **A**–**C** Estimated relationship between tick abundances and monthly climate predictors: **A** maximum temperature, **B** specific humidity, **C** PDSI (higher values, less drought). For each panel, the* x*-axis represents the standardized values of the predictor variables, and the* y*-axis shows the estimated effect on tick abundances (on the log_10_ scale). Asterisks (*) denote the significance level of the climate variable interaction switch climate region at *P < 0.1, **P < 0.01 and ***P < 0.001. CC, Central Coast; SF, San Francisco; LA, Los Angeles; PSDI, Palmer Drought Severity Index

To investigate how juvenile tick abundances are influenced by climate, we fit GAMs to our juvenile abundances on individual lizards per sampling event (location-month-year). Our model for total tick abundances did not show significant linear relationships with climate regions, but it did reveal important interactions between climate regions and abiotic variables through the smoothing terms (Fig. 3). Notably, the interaction with monthly maximum temperature showed a significant negative relationship with tick abundances: in the San Francisco region, tick abundances decreased as temperature rose above the regional mean (*P* = 0.02), while in Los Angeles, tick abundances were negatively associated with temperature but followed a stronger, linear pattern (*P* = 0.004). For San Francisco, the relationship was slightly nonlinear (e*df* = 3.0), with a sharp increase in tick abundances below the mean temperature, followed by a gradual decline above the mean. In contrast, the Los Angeles region exhibited a strongly linear relationship (*edf* = 1.0) where higher maximum temperatures led to lower tick abundances.

In addition, we found a significant positive effect of monthly specific humidity on tick abundances in the Los Angeles region (*P* < 0.001), whereas the Central Coast region showed an opposite trend, with tick abundances declining as humidity exceeded the regional mean (*P* = 0.003). Furthermore, the relationship between drought conditions (measured by the PDSI) and tick abundances was generally positive, with less drought (higher PDSI values) associated with greater tick abundances. However, this relationship was only statistically significant in Los Angeles (*P* = 0.002). Our total tick abundances model also accounted for spatial autocorrelation using a smoothing term for geographic location, which was highly significant (*P* < 0.001). Overall, the model explained 56.4% of the deviance, with an adjusted* R*^2^ of 47.2%. The model’s REML value was 3531.3, with a scale estimate of 3.38, and was based on 1,527 observations. Model diagnostics can be found in Additional file 5: Figure S1.

Separate models for larval and nymphal abundances failed to converge when the spatial autocorrelation term (s(lon, lat, bs = “gp”)) was included but were successfully fitted with the other smoothing interaction terms. Results from these models are shown in Additional file 5: Figure S2.

## Discussion

Our study reveals significant variation in juvenile tick phenology across California, suggesting that climate-driven changes in juvenile tick host-seeking behavior may contribute to regional differences in Lyme disease prevalence in ticks. By using lizards—the preferred host for juvenile *I. pacificus* ticks [27, 28]—instead of traditional tick drags, we increased detection probability in areas with lower tick densities. This approach helps overcome previous challenges in detecting off-host juvenile ticks across the significant latitudinal gradient where this species is distributed. This study represents one of the most extensive spatial analyses of juvenile *I. pacificus* in California, providing valuable insights that could inform vector surveillance and improve our understanding of Lyme disease risk across a climatically diverse landscape.

We observed substantial variability in mean juvenile tick abundances on lizards, ranging from 0.17 to 47.21, with the highest mean abundances found in the San Francisco Bay Area and the lowest mean abundances in the Los Angeles region. Notably, in the San Francisco Bay Area, nymphs peaked 25 days earlier than larvae, a pattern not observed in the Central Coast and Los Angeles regions, where larvae and nymphs reached peak mean abundance on the same Julian date for each respective region. The earlier peak in nymphal abundances in the San Francisco Bay Area may increase the potential for Lyme disease transmission, as infectious nymphs could infect the rodent host population which will host the larvae that emerge later, amplifying infection rates [55]. Such a timing difference in tick phenology may explain the higher Lyme disease prevalence in ticks observed in the San Francisco Bay Area, highlighting the importance of temporal dynamics in pathogen transmission [5, 33, 52, 56–58]. A number of ecological hypotheses suggest that this pattern may emerge in the San Francisco Bay Area, rather than in the Central Coast or Los Angeles regions, due to the generally lower annual mean temperatures [22]. These cooler conditions may allow juvenile ticks to extend their seasonal activity, enabling larvae to reach peak abundances later in the year compared to those in drier areas, where increased desiccation may prevent later peaks [59]. Alternatively, juvenile abundances are higher in northern California than in southern California, which may enhance our ability to detect more pronounced differences in the seasonal activity of juvenile *I. pacificus* [22].

We also quantified the overlap between larval and nymphal populations and observed a clear latitudinal trend in this overlap. The highest overlap occurred in the San Francisco Bay Area, which was approximately twofold higher than that in the Central Coast region and approximately threefold higher than that in the Los Angeles region. This finding contradicted our initial hypothesis that we would observe the highest juvenile overlap in warmer regions, such as Los Angeles, where a shorter season might intensify overlap due to an accelerated tick life-cycle [59]. However, this unexpected pattern may be influenced by the timing of our sampling in relation to tick emergence (Additional file 1: Table S2) and perhaps a longer season in northern than southern California [59]. Yet prior studies in Mendocino, in northwestern California, suggest similar temporal patterns of juvenile overlap as our San Francisco Bay Area results [9, 60, 61]. There is also the possibility that nymphs in the San Francisco Bay Area exhibit bimodal seasonality, while larvae follow a unimodal pattern [63]. This could explain our findings that the San Francisco Bay Area shows the highest degree of overlap (when one portion of nymphs coincide with the larvae) as well as the greatest separation of life stages (when the other portion of nymphs emerge outside the unimodal larval peak) [63]. A more systematic sampling design, spanning pre- and post-season periods, would better capture these dynamics and provide a clearer picture of how overlap varies across regions. Additionally, including data from more northern regions of the state, such as Mendocino County, could have provided a clearer contrast to the southern areas, given its higher precipitation, larger tick populations, and elevated risk of Lyme disease. Finally, we found that in months with higher maximum temperature and with more severe drought conditions, tick abundances on lizards were lower (Fig. 3; Table 2). While these findings are consistent with previously documented relationships, we extend this understanding by demonstrating that both the shape and magnitude of these relationships vary across different climate regions [9, 19, 47, 62, 63].

**Table 2:** Generalized additive models predicting log_10_-transformed juvenile tick abundance on individual lizards with climate variables standardized

Outcome: total juvenile abundances per lizard					
Linear terms	Predictors^a,b^	Estimates	SE	*t*-value	*P*-value
	SF (intercept)	2.72	5.79	0.47	0.64
	CC	− 0.29	7.05	− 0.041	0.97
	LA	− 3.82	8.40	− 0.46	0.69

Smooth terms	Predictors^a,b^	*edf*	Reference* df*	*F*	*P*-value
	tmmx–SF	3.084	3.76	2.93	0.018*
	tmmx–CC	1.0	1.00	2.044	0.15
	tmmx–LA	1.0	1.00	8.58	0.0034**
	sph–SF	1.0	1.00	0.76	0.38
	sph–CC	4.41	5.28	3.61	0.0025**
	sph–LA	4.27	5.013	5.94	< 0.001***
	pdsi–SF	2.17	2.71	1.56	0.29937
	pdsi–CC	1.00	1.00	0.033	0.86
	pdsi–LA	3.54	4.13	4.43	0.0023**
	Lon, Lat	26.00	27.59	14.21	< 0.001***

Tweedie distribution with a log link function

Observations = 1527.* R*^2^(adj) = 0.472. Deviance explained = 56.4%. REML = 3534.8; scale est 3.3677

*edf* Estimated degrees of freedom,* SE* standard error

*, **, ***Significant at *P < 0.1, **P < 0.01, ***P < 0.001

^a^ Climate regions: SF, San Francisco; CC, Central Coast, LA, Los Angeles

^b^Monthly climate variables: tmmx, maximum temperature; sph, specific humidity (sph); PDSI, Palmer Drought Severity Index (drought index)

Our results confirm that tick phenology patterns are latitudinally structured, with tick abundances highest in cooler northern regions compared to warmer southern regions [17, 59, 64]. However, this simple north-to-south gradient narrative overlooks the complexity of tick seasonality, especially in a state with a large coastal climate influence that overlaps many established tick populations [41, 44, 65]. Additional factors, such as humidity, which is influenced by proximity to the coast, can extend the tick season, as observed in the Central Coast region (Fig. 2) [19, 44]. When examining the effect of humidity on total tick abundances, we found a strong positive relationship in the Los Angeles region, which is typically drier than Central California, suggesting that tick abundances in this region may be limited by moisture in the environment. In contrast, humidity above the regional mean decreased tick abundances in the Central California region, possibly because it surpassed the physiological tolerance threshold for juvenile ticks [19, 59]. While we observed that tick activity starts earliest in the Central Coast region, followed by Los Angeles and finally San Francisco Bay Area, this temporal pattern may reflect sampling design rather than underlying ecological differences (Additional file 1: Table S2). Although habitat suitability factors like elevation and forest cover may account for additional variation in our climate models, we believe that these habitat variables serve as proxies for humidity and temperature, particularly in California’s far western region [17, 66, 67].

An intriguing finding, although potentially influenced by sampling methods, was the earlier peak of nymphs compared to larvae in the San Francisco Bay Area. In California, it is generally assumed that larvae emerge at the same time as, or before nymphs, which may limit the transmission potential of the Lyme disease agent, as larvae can only acquire the bacterium through an infectious blood meal [68]. In our study, however, nymphal abundances peaked 25 days prior to the larval peak. If this earlier peak in nymphs is accurate, it could indicate that more vertebrates are exposed to infectious blood meals before larvae emerge. An intriguing follow-up study could explore the minimum time period or proportion of nymphs that need to feed before larvae in order to significantly increase the presence of *B. burgdorferi* in rodents. We also recognize that lizards do not directly provide infectious blood meals, but they may serve as proxies for broader juvenile tick activity patterns, potentially reflecting rodent tick burden dynamics [27–29].

If lizard burdens correlate with general tick burdens on pathogen-amplifying rodents, our model results suggest that climate change could influence tick-borne pathogen transmission dynamics in California. Our climate models indicate that tick burdens are highest under cooler monthly temperatures, optimal humidity levels and lower drought stress. As northern regions of the state warm due to climate change, we may see a latitudinal shift in phenology and transmission patterns. However, relative humidity plays a crucial role, and ticks in humid coastal areas may find refuge in the more temperate southern coastal regions [19, 31]. Additionally, while drought conditions are widespread across California, their impact on tick populations will depend on the intensity and duration of the droughts. In warmer, drier areas, more severe droughts are likely to cause a decline in tick populations [59]. Our results suggest that in regions with optimal climate conditions that support longer within-season activity, larger tick populations may drive higher transmission rates. In contrast, in suboptimal areas with shorter activity seasons and reduced tick populations, opportunities for juvenile ticks to interact with pathogen-amplifying rodent hosts are limited. Nonetheless, even in these suboptimal conditions, ticks can seek refuge in microclimates, allowing them to persist and still contribute to pathogen amplification, although likely at a reduced rate.

While tick abundances on lizards may provide a more accurate method for estimating juvenile *I. pacificus* population dynamics compared to traditional drag cloth sampling, there are opportunities to refine this approach. Future studies should collect additional data on population density of lizards as well as characteristics of individual lizards, such as snout-to-vent length and sex, to better standardize results across different individuals, as these factors may influence total tick abundances [21, 24, 69]. Our dataset included two primary species of lizards: alligator lizards and western fence lizards. While differences in size between these species could potentially introduce biases in tick averages, most alligator lizards were captured in southern California, where they exhibited, on average, lower tick burdens compared to their northern counterparts. Interestingly, these latitudinal trends in tick burdens closely mirrored those observed in western fence lizards. In the eastern USA, variation in host-seeking behavior of *I. scapularis* ticks has been hypothesized to be driven by evolutionary differences in thermal tolerance between southern and northern populations. A similar mechanism may be influencing the behavior of *I. pacificus* populations, which could be explored through future molecular studies [14, 23]. We acknowledge that repeated sampling at a single location, combined with data loggers (e.g., HOBO loggers), represents the gold standard for linking microclimatic conditions to tick seasonality. However, due to the time and logistical constraints associated with conducting such studies across multiple locations with diverse climate gradients, this approach may not be feasible for many research teams. We, therefore, recommend that future research incorporates unbiased data alongside the important metadata mentioned above, which will facilitate the synthesis of disparate ecological datasets to address broader ecological questions related to tick behavior and its implications for human health.

## Conclusions

This study provides new insights into the regional variation in juvenile *I. pacificus* phenology across California, revealing climate-driven differences in tick abundances that may help explain regional Lyme disease prevalence. Our findings suggest that factors such as temperature, coastal humidity and drought severity influence tick seasonality, with notable differences in tick activity patterns across regions, particularly between the San Francisco Bay Area and Los Angeles regions. The earlier emergence of nymphs compared to larvae in the San Francisco Bay Area could increase the potential for Lyme disease transmission, which is consistent with variation in reported human incidence and epidemiological studies and warrants further investigation. By comparing data across a broad set latitudinal gradient, we offer valuable insights into juvenile tick dynamics and emphasize the need for continued longitudinal research to refine our understanding and inform vector control strategies.

## Supplementary Information

Supplementary Material 1.

Supplementary Material 2.

Supplementary Material 3.

Supplementary Material 4.

Supplementary Material 5.

## Data Availability

The datasets supporting the conclusions of this article as well as code for the figures and analysis are available in a Dryad digital repository (10.5061/dryad.v6wwpzh67) and a GitHub repository (https://github.com/sbsambado/ca_lizardburden).

## Abbreviations

CDD: Cumulative degree days
CC: Central Coast region
GAMs: Generalized additive models
JI: Jaccard index or Jaccard similarity coefficient
KDE: Kernel density estimation
LA: Los Angeles region
SF: San Francisco Bay Area region
