# Supplementary material for: Climate-driven variation in the phenology of juvenile Ixodes pacificus on lizard hosts
Source: Parasit Vectors. 2025 Apr 15;18:141. doi: 10.1186/s13071-025-06749-4 (PMC12001419; doi:10.1186/s13071-025-06749-4)
Supplement: Supplementary file 1 — Supplementary Material 1. [file 13071_2025_6749_MOESM1_ESM.docx]

**Climate-driven variation in the phenology of juvenile *Ixodes pacificus* on lizard hosts**

**Samantha Sambado^1*^, Amanda Sparkman^2^, Andrea Swei^3^, Andrew J MacDonald^4^, Hillary S Young^1^, Jordan Salomon^5^, Arielle Crews^6^, Kacie Ring^1^, Stephanie Copeland^1^, and Cheryl J Briggs^1^**

1. Ecology, Evolution & Marine Biology Department at University of California Santa Barbara, Santa Barbara, California, USA

2. Biology Department at Westmont College, Santa Barbara, California, USA

3. Biology Department at San Francisco State University, California, USA

4. Bren School of Environmental Science & Management at University of California Santa Barbara, California, USA

5. Ecology & Evolutionary Biology Program at Texas A&M University, College Station, Texas, USA

6. San Mateo County Mosquito and Vector Control, Burlingame, California, USA

***Correspondence**: [sbsambado@ucsb.edu](mailto:sbsambado@ucsb.edu)

**Supplementary information**

TABLE OF CONTENTS

**Additional file 1: Sampling locations**

**Table S1.** Location coordinates

**Table S2.** Location sample dates

**Additional file 2: Location characteristics**

**Figure S1.** Location sampling frequency

**Figure S2.** Lizards

**Table S1.** mean and sd per location

**Additional file 3: Method details**

**Text S1.** Additional details on Field methods

**Text S2.** Statistical method justifications

**Figure S1.** Covariate correlation and vif results

**Additional file 4: Phenological metrics by climate regions**

**Figure S1.** Distribution of juvenile burdens by CR3

**Figure S2.** Ticks per month and year by CR5

**Table S1.** Phenology metrics for all climate regions

**Additional file 5: GAM results and diagnostics**

**Figure S1.** GAM 1 results and diagnostics

**Figure S2.** GAM 2 results and diagnostics

**ADDITIONAL FILE 1: Sampling locations**

**Additional file 1: Table S1.** Field sampling locations, including collector,  geographic coordinates, and climate region (CR).

| **CR** | **Location** | **Collector** | **Latitude** | **Longitude** |
| --- | --- | --- | --- | --- |
| North Coast | McLaughlin | Sambado | 38.87289 | -122.43265 |
| San Francisco Bay Area | Quail Ridge | Sambado | 38.48899 | -122.15171 |
| San Francisco Bay Area | SLRP | Swei | 38.42810 | -122.62120 |
| San Francisco Bay Area | SMI | Swei | 38.01881 | -122.57748 |
| San Francisco Bay Area | CCSP | Swei | 38.00110 | -122.48930 |
| San Francisco Bay Area | TIL | Swei | 37.89770 | -122.24520 |
| San Francisco Bay Area | LFY | Swei | 37.87900 | -122.14240 |
| San Francisco Bay Area | SLRP | Swei | 37.87900 | -122.14240 |
| San Francisco Bay Area | PR | Swei | 37.61770 | -121.88970 |
| San Francisco Bay Area | San Mateo | Sparkman | 37.55417 | -122.31306 |
| San Francisco Bay Area | WDLP | Swei | 37.50610 | -122.30260 |
| San Francisco Bay Area | PUG | Swei | 37.47870 | -122.29220 |
| San Francisco Bay Area | FL | Swei | 37.47188 | -122.31080 |
| San Francisco Bay Area | WH | Swei | 37.36250 | -122.23150 |
| San Francisco Bay Area | HOS | Swei | 37.23260 | -121.92940 |
| San Francisco Bay Area | WP | Swei | 37.22210 | -121.96650 |
| San Francisco Bay Area | San Mateo | Sparkman | 37.16939 | -121.98051 |
| San Francisco Bay Area | San Mateo | Sparkman | 37.16860 | -121.91160 |
| Central Coast | Fort Ord | Sambado | 36.68491 | -121.77692 |
| Central Coast | Hastings | Sambado | 36.38845 | -121.54774 |
| Central Coast | Big Creek | Sambado | 36.07016 | -121.59829 |
| Central Coast | Rancho Marino | Sambado | 35.53549 | -121.08241 |
| Central Coast | San Luis Obispo | Sparkman | 35.31727 | -120.64722 |
| Central Coast | San Luis Obispo | Sparkman | 35.30500 | -120.66250 |
| San Joaquin Valley | Arid | Young | 35.02487 | -118.67928 |
| San Joaquin Valley | Intermediate | Young | 34.97189 | -118.59146 |
| San Joaquin Valley | Mesic | Young | 34.97189 | -118.58362 |
| Central Coast | Sedgwick | Sambado | 34.69812 | -120.04715 |
| Central Coast | Sedgwick Reserve | Sparkman | 34.69390 | -120.04170 |
| Central Coast | Paradise Rd unburn | MacDonald | 34.55173 | -119.77865 |
| Central Coast | Santa Barbara | Sparkman | 34.54694 | -119.69097 |
| Central Coast | Santa Barbara | Sparkman | 34.47466 | -119.70511 |
| Central Coast | Santa Barbara | Sparkman | 34.47110 | -119.68610 |
| Central Coast | Santa Barbara | Sparkman | 34.45916 | -119.65430 |
| Central Coast | Santa Barbara | Sparkman | 34.45083 | -119.67875 |
| Central Coast | Santa Barbara | Sparkman | 34.44880 | -119.66100 |
| Central Coast | Santa Barbara | Sparkman | 34.44388 | -119.59457 |
| Central Coast | Santa Barbara | Sparkman | 34.42083 | -119.69819 |
| Central Coast | Santa Barbara | Sparkman | 34.42080 | -119.69820 |
| Central Coast | Coal Oil Point | Sambado | 34.41630 | -119.87790 |
| Los Angeles | Santa Monica Mountains | Sparkman | 34.12030 | -118.93180 |
| Los Angeles | Stunt Ranch | Sambado | 34.09381 | -118.65601 |
| Los Angeles | Santa Cruz Island | Sparkman | 34.02320 | -119.76580 |
| Los Angeles | Santa Cruz Island | Sambado | 34.00138 | -119.71451 |
| Los Angeles | Santa Rosa Island | Sparkman | 33.97730 | -120.08960 |

**Additional file 1: Table S2.** The number of lizards sampled per location, year, and month. Climate regions (CR) area: NC = North Coast, SF = San Francisco Bay Area, CC = Central Coast, SJV = San Joaquin Valley, LA = Los Angeles. ‘NA’ refers to sites that were not sampled, while ‘0’ refers to sites that were sampled but no lizards were spotted and/or captured.

| **Region** | **Location** | **Year** | **Feb.** | **March** | **April** | **May** | **June** |
| --- | --- | --- | --- | --- | --- | --- | --- |
| NC | McLaughlin | 2021 | NA | 0 | 6 | 4 | 2 |
| SF | Quail Ridge | 2021 | NA | 0 | 4 | 2 | 2 |
| SF | SLRP | 2018 | NA | NA | 10 | NA | NA |
| SF | SMI | 2018 | NA | NA | 1 | NA | NA |
| SF | SMI | 2019 | NA | NA | 11 | 3 | NA |
| SF | CCSP | 2018 | NA | NA | 18 | NA | NA |
| SF | CCSP | 2019 | NA | NA | 16 | NA | NA |
| SF | TIL | 2019 | NA | NA | NA | 5 | NA |
| SF | LFY | 2018 | NA | NA | 15 | NA | NA |
| SF | LFY | 2019 | NA | NA | NA | NA | 16 |
| SF | SLRP | 2019 | NA | NA | NA | 22 | NA |
| SF | PR | 2019 | NA | NA | NA | 5 | NA |
| SF | San Mateo | 2015 | NA | NA | NA | NA | 10 |
| SF | San Mateo | 2016 | NA | NA | NA | 9 | NA |
| SF | San Mateo | 2017 | NA | NA | NA | 23 | 2 |
| SF | San Mateo | 2018 | NA | NA | NA | 13 | 2 |
| SF | San Mateo | 2019 | NA | NA | NA | 5 | NA |
| SF | San Mateo | 2021 | NA | NA | NA | 7 | NA |
| SF | San Mateo | 2022 | NA | NA | NA | 14 | NA |
| SF | WDLP | 2018 | NA | NA | 9 | NA | NA |
| SF | WDLP | 2019 | NA | NA | NA | 3 | NA |
| SF | PUG | 2019 | NA | NA | 15 | NA | NA |
| SF | FL | 2018 | NA | NA | 4 | NA | NA |
| SF | FL | 2019 | NA | NA | NA | 3 | NA |
| SF | WH | 2018 | NA | NA | 15 | NA | NA |
| SF | WH | 2019 | NA | NA | 17 | NA | NA |
| SF | HOS | 2018 | NA | NA | NA | 17 | NA |
| SF | HOS | 2019 | NA | NA | 17 | NA | NA |
| SF | WP | 2018 | NA | NA | NA | 16 | NA |
| SF | WP | 2019 | NA | NA | 15 | NA | NA |
| SF | San Mateo | 2018 | NA | NA | NA | 1 | NA |
| SF | San Mateo | 2018 | NA | NA | NA | 1 | NA |
| CC | Fort Ord | 2021 | NA | 0 | 2 | 15 | 15 |
| CC | Hastings | 2021 | NA | 0 | 7 | 15 | 15 |
| CC | Big Creek | 2021 | NA | 0 | 12 | 15 | 15 |
| CC | Rancho Marino | 2021 | NA | 0 | 15 | 15 | 15 |
| CC | San Luis Obispo | 2019 | NA | NA | NA | 10 | NA |
| CC | San Luis Obispo | 2019 | NA | NA | NA | 11 | NA |
| SJ | Arid | 2018 | NA | NA | 33 | NA | NA |
| SJ | Intermediate | 2018 | NA | NA | NA | 62 | NA |
| SJ | Mesic | 2018 | NA | NA | NA | 2 | NA |
| CC | Sedgwick | 2021 | NA | 8 | 9 | 8 | 12 |
| CC | Sedgwick Reserve | 2021 | NA | 4 | NA | 3 | NA |
| CC | Sedgwick Reserve | 2022 | NA | NA | 2 | NA | NA |
| CC | Paradise Rd unburn | 2014 | 11 | 13 | 25 | 10 | NA |
| CC | Santa Barbara | 2019 | NA | NA | NA | 2 | NA |
| CC | Santa Barbara | 2019 | NA | NA | NA | NA | 13 |
| CC | Santa Barbara | 2021 | NA | NA | NA | NA | 8 |
| CC | Santa Barbara | 2022 | NA | NA | NA | NA | 1 |
| CC | Santa Barbara | 2018 | NA | NA | NA | 23 | NA |
| CC | Santa Barbara | 2019 | NA | NA | NA | 6 | 11 |
| CC | Santa Barbara | 2021 | NA | NA | NA | NA | 7 |
| CC | Santa Barbara | 2022 | NA | NA | NA | NA | 3 |
| CC | Santa Barbara | 2022 | NA | NA | NA | NA | 2 |
| CC | Santa Barbara | 2019 | NA | NA | NA | 13 | NA |
| CC | Santa Barbara | 2022 | NA | NA | NA | NA | 2 |
| CC | Santa Barbara | 2019 | NA | NA | NA | 34 | 9 |
| CC | Santa Barbara | 2021 | NA | NA | NA | 7 | 12 |
| CC | Santa Barbara | 2021 | NA | NA | NA | NA | 1 |
| CC | Santa Barbara | 2022 | NA | NA | NA | NA | 2 |
| CC | Santa Barbara | 2014 | NA | NA | 3 | 1 | NA |
| CC | Santa Barbara | 2016 | NA | 1 | NA | NA | NA |
| CC | Santa Barbara | 2018 | NA | 2 | NA | 3 | NA |
| CC | Santa Barbara | 2019 | NA | NA | NA | 9 | 1 |
| CC | Santa Barbara | 2021 | NA | NA | NA | 1 | 1 |
| CC | Coal Oil Point | 2021 | NA | 1 | 1 | 8 | 2 |
| LA | Santa Monica Mts. | 2014 | NA | NA | NA | 6 | NA |
| LA | Santa Monica Mts. | 2017 | NA | NA | NA | 1 | NA |
| LA | Stunt Ranch | 2021 | NA | 9 | 15 | 12 | 15 |
| LA | Santa Cruz Island | 2013 | NA | NA | NA | NA | 4 |
| LA | Santa Cruz Island | 2014 | NA | NA | NA | 8 | 13 |
| LA | Santa Cruz Island | 2015 | NA | 28 | NA | 17 | NA |
| LA | Santa Cruz Island | 2016 | NA | 26 | NA | 43 | NA |
| LA | Santa Cruz Island | 2017 | NA | 4 | NA | 66 | NA |
| LA | Santa Cruz Island | 2018 | NA | 15 | NA | 17 | 4 |
| LA | Santa Cruz Island | 2019 | NA | 44 | NA | 29 | NA |
| LA | Santa Cruz Island | 2021 | NA | 11 | NA | 14 | NA |
| LA | Santa Cruz Island | 2022 | NA | 6 | NA | 6 | NA |
| LA | Santa Cruz Island | 2021 | NA | NA | 3 | 8 | 9 |
| LA | Santa Rosa Island | 2015 | NA | NA | NA | 16 | NA |
| LA | Santa Rosa Island | 2016 | NA | NA | 20 | 5 | NA |
| LA | Santa Rosa Island | 2018 | NA | 2 | NA | NA | NA |
| LA | Santa Rosa Island | 2019 | NA | 8 | 3 | NA | NA |
| LA | Santa Rosa Island | 2021 | NA | 23 | NA | 34 | NA |
| LA | Santa Rosa Island | 2022 | NA | 28 | NA | 22 | NA |
