# Supplementary material for: Climate-driven variation in the phenology of juvenile Ixodes pacificus on lizard hosts
Source: Parasit Vectors. 2025 Apr 15;18:141. doi: 10.1186/s13071-025-06749-4 (PMC12001419; doi:10.1186/s13071-025-06749-4)
Supplement: Supplementary file 2 — Supplementary Material 2. [file 13071_2025_6749_MOESM2_ESM.docx]

**Climate-driven variation in the phenology of juvenile *Ixodes pacificus* on lizard hosts**

**Samantha Sambado^1*^, Amanda Sparkman^2^, Andrea Swei^3^, Andrew J MacDonald^4^, Hillary S Young^1^, Jordan Salomon^5^, Arielle Crews^6^, Kacie Ring^1^, Stephanie Copeland^1^, and Cheryl J Briggs^1^**

1. Ecology, Evolution & Marine Biology Department at University of California Santa Barbara, Santa Barbara, California, USA

2. Biology Department at Westmont College, Santa Barbara, California, USA

3. Biology Department at San Francisco State University, California, USA

4. Bren School of Environmental Science & Management at University of California Santa Barbara, California, USA

5. Ecology & Evolutionary Biology Program at Texas A&M University, College Station, Texas, USA

6. San Mateo County Mosquito and Vector Control, Burlingame, California, USA

***Correspondence**: [sbsambado@ucsb.edu](mailto:sbsambado@ucsb.edu)

**Supplementary information**

TABLE OF CONTENTS

**Additional file 1: Sampling locations**

**Table S1.** Location coordinates

**Table S2.** Location sample dates

**Additional file 2: Location characteristics**

**Figure S1.** Location sampling frequency

**Figure S2.** Lizards

**Table S1.** mean and sd per location

**Additional file 3: Method details**

**Text S1.** Additional details on Field methods

**Text S2.** Statistical method justifications

**Figure S1.** Covariate correlation and vif results

**Additional file 4: Phenological metrics by climate regions**

**Figure S1.** Distribution of juvenile burdens by CR3

**Figure S2.** Ticks per month and year by CR5

**Table S1.** Phenology metrics for all climate regions

**Additional file 5: GAM results and diagnostics**

**Figure S1.** GAM 1 results and diagnostics

**Figure S2.** GAM 2 results and diagnostics

**ADDITIONAL FILE 2: Location characteristics**

**Additional file 2: Figure S1.** Bar graph displaying the frequency of visits to various locations, colored by climatic region and arranged from northern to southern latitude. The black dashed line indicates locations that were visited at least three times. Santa Cruz (SC) Island was sampled separately by two collaborators: Sambado (SS) and Sparkman (AS).


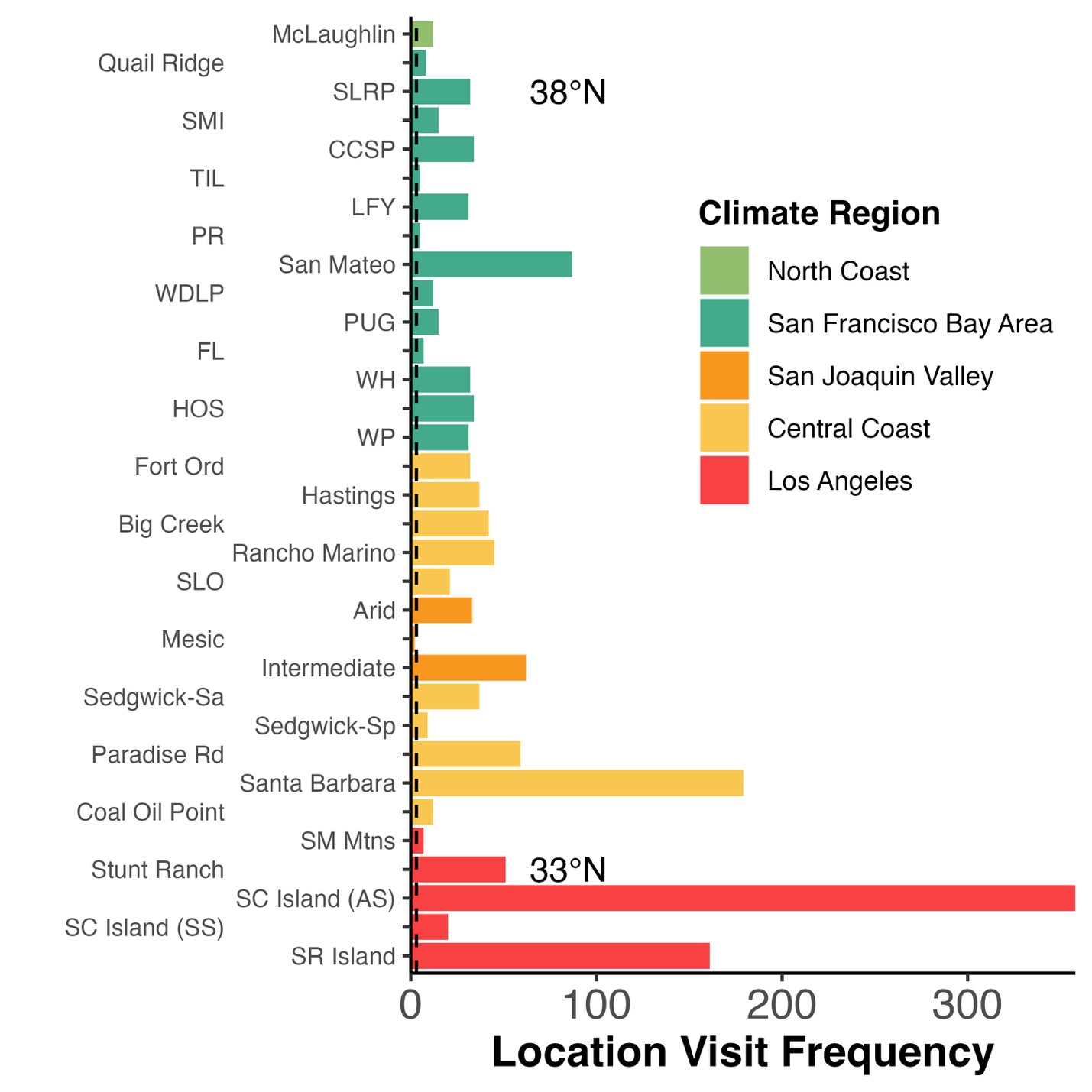


**Additional file 2: Figure S2.** Box plots illustrating the number of lizards sampled at each location during individual sampling dates. The colors of the box plots correspond to different climatic regions. Santa Cruz (SC) Island was sampled separately by two collaborators: Sambado (SS) and Sparkman (AS).


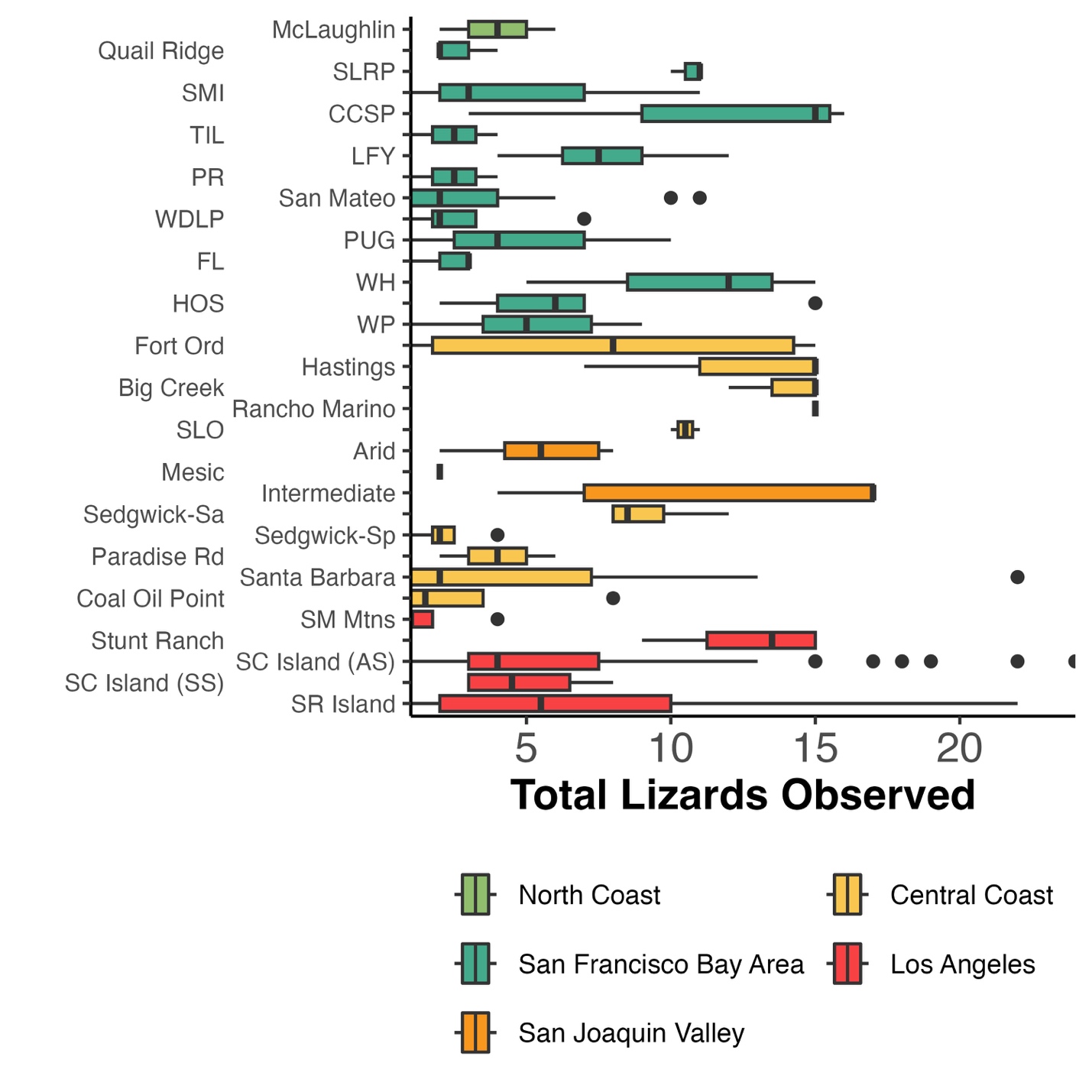


**Additional file 2: Table S1.** Mean and standard deviation (sd) of abundance for all ticks, larval ticks, and nymphal ticks per individual lizard, per location.

| **LOCATION** | **TOTAL TICKS** | | **LARVAE** | | **NYMPHS** | |
| --- | --- | --- | --- | --- | --- | --- |
|  | **Mean** | **SD** | **Mean** | **SD** | **Mean** | **SD** |
| McLaughlin | 6.833 | 6.394 | 2.583 | 3.579 | 4.25 | 5.61 |
| Quail Ridge | 11 | 9.258 | 3.75 | 2.866 | 7.125 | 7.06 |
| SLRP | 30.9 | 30.113 | 17.8 | 31.538 | 12.7 | 8.028 |
| SMI | 7.8 | 7.58 | 0.467 | 0.516 | 7.333 | 7.761 |
| CCSP | 47.206 | 33.462 | 25.324 | 27.377 | 21.882 | 13.137 |
| TIL | 31.4 | 26.605 | 21 | 25.328 | 10.4 | 5.595 |
| LFY | 11.774 | 9.014 | 8.871 | 7.877 | 2.903 | 2.534 |
| SLRP | 15.591 | 12.339 | 7.636 | 9.801 | 7.955 | 6.137 |
| PR | 5.4 | 0.894 | 4.6 | 1.342 | 0.8 | 0.447 |
| San Mateo | 7.741 | 8.156 | NA | NA | NA | NA |
| WDLP | 19.75 | 15.662 | 11.417 | 12.288 | 8.333 | 5.71 |
| PUG | 4.333 | 7.49 | 2.6 | 6.367 | 1.733 | 2.086 |
| FL | 29.714 | 19.872 | 22.714 | 18.09 | 7 | 5.292 |
| WH | 30.062 | 26.109 | 12.906 | 16.528 | 17.156 | 14.065 |
| HOS | 32.559 | 30.348 | 23.059 | 25.943 | 9.5 | 7.867 |
| WP | 4.645 | 4.765 | 3.194 | 3.728 | 1.452 | 1.69 |
| San Mateo | 5 | NA | NA | NA | NA | NA |
| San Mateo | 5 | NA | NA | NA | NA | NA |
| Fort Ord | 8 | 9.449 | 6.281 | 7.924 | 1.719 | 2.02 |
| Hastings | 4.919 | 8.2 | 2.703 | 5.821 | 2.216 | 3.065 |
| Big Creek | 1.643 | 2.151 | 0.619 | 1.011 | 1.048 | 1.652 |
| Rancho Marino | 12.2 | 11.565 | 5.333 | 7.173 | 6.867 | 5.983 |
| San Luis Obispo | 5.8 | 4.315 | NA | NA | NA | NA |
| San Luis Obispo | 0.455 | 0.688 | NA | NA | NA | NA |
| Arid | 0.545 | 1.46 | NA | NA | NA | NA |
| Intermediate | 1.113 | 2.025 | NA | NA | NA | NA |
| Mesic | 1 | 1.414 | NA | NA | NA | NA |
| Sedgwick | 4.405 | 5.166 | 1.622 | 3.277 | 2.784 | 3.787 |
| Sedgwick Reserve | 3.111 | 4.137 | NA | NA | NA | NA |
| Paradise Rd unburn | 4.932 | 4.164 | 4.034 | 3.429 | 0.898 | 1.078 |
| Santa Barbara | 5.5 | 2.121 | NA | NA | NA | NA |
| Santa Barbara | 0.636 | 0.902 | NA | NA | NA | NA |
| Santa Barbara | 1.4 | 2.185 | NA | NA | NA | NA |
| Santa Barbara | 1 | 0 | NA | NA | NA | NA |
| Santa Barbara | 1.8 | 2.541 | NA | NA | NA | NA |
| Santa Barbara | 0 | 0 | NA | NA | NA | NA |
| Santa Barbara | 1 | NA | NA | NA | NA | NA |
| Santa Barbara | 1 | 0 | NA | NA | NA | NA |
| Santa Barbara | 2.696 | 4.527 | NA | NA | NA | NA |
| Coal Oil Point | 0.167 | 0.389 | 0 | 0 | 0.167 | 0.389 |
| Santa Monica Mnts | 3.714 | 2.628 | NA | NA | NA | NA |
| Stunt Ranch | 1.627 | 2.039 | 0.353 | 0.996 | 1.275 | 1.65 |
| Santa Cruz Island | 2.109 | 4.806 | NA | NA | NA | NA |
| Santa Cruz Island | 1.45 | 2.089 | 0.25 | 0.55 | 1.2 | 1.963 |
| Santa Rosa Island | 0.497 | 1.848 | NA | NA | NA | NA |
