# Supplementary material for: Climate-driven variation in the phenology of juvenile Ixodes pacificus on lizard hosts
Source: Parasit Vectors. 2025 Apr 15;18:141. doi: 10.1186/s13071-025-06749-4 (PMC12001419; doi:10.1186/s13071-025-06749-4)

**Climate-driven variation in the phenology of juvenile *Ixodes pacificus* on lizard hosts**

**Samantha Sambado^1*^, Amanda Sparkman^2^, Andrea Swei^3^, Andrew J MacDonald^4^, Hillary S Young^1^, Jordan Salomon^5^, Arielle Crews^6^, Kacie Ring^1^, Stephanie Copeland^1^, and Cheryl J Briggs^1^**

1. Ecology, Evolution & Marine Biology Department at University of California Santa Barbara, Santa Barbara, California, USA

2. Biology Department at Westmont College, Santa Barbara, California, USA

3. Biology Department at San Francisco State University, California, USA

4. Bren School of Environmental Science & Management at University of California Santa Barbara, California, USA

5. Ecology & Evolutionary Biology Program at Texas A&M University, College Station, Texas, USA

6. San Mateo County Mosquito and Vector Control, Burlingame, California, USA

***Correspondence**: [sbsambado@ucsb.edu](mailto:sbsambado@ucsb.edu)

**Supplementary information**

TABLE OF CONTENTS

**Additional file 1: Sampling locations**

**Table S1.** Location coordinates

**Table S2.** Location sample dates

**Additional file 2: Location characteristics**

**Figure S1.** Location sampling frequency

**Figure S2.** Lizards

**Table S1.** mean and sd per location

**Additional file 3: Method details**

**Text S1.** Additional details on Field methods

**Text S2.** Statistical method justifications

**Figure S1.** Covariate correlation and vif results

**Additional file 4: Phenological metrics by climate regions**

**Figure S1.** Distribution of juvenile burdens by CR3

**Figure S2.** Ticks per month and year by CR5

**Table S1.** Phenology metrics for all climate regions

**Additional file 5: GAM results and diagnostics**

**Figure S1.** GAM 1 results and diagnostics

**Figure S2.** GAM 2 results and diagnostics

**ADDITIONAL FILE 3: Method details**

**Additional file 3: Text S1. Additional details on field methods**

For Sambado data:

Lizard surveys began after 11:00 AM at each plot. Western fence lizards (*Sceloporus*

*occidentalis*) were captured using a 0.5 m fishing pole with a dental floss lasso at the tip (1). Each plot was surveyed for a duration of twenty minutes. When a lizard was captured, the timer was paused and resumed upon the lizard’s release at the capture site. Tick burdens were assessed on the ears and nuchal pouches. To prevent double sampling of individuals within a month, white-out paint was applied to the lizard's back. Each month, the paint was applied to a specific body part to denote new collections: March - head, April - neck, May - midback, and June - tail. If no lizards were observed during the 20-minute survey, additional searching was conducted off the plot in areas with more suitable habitat (e.g., fallen logs, exposed rocks, near cover boards) for another 10 minutes within a 100 m radius.

For Sparkman data:

Western fence lizards (*Sceloporus occidentalis*), southern alligator lizards (*Elgaria multicarinata*), and side-blotched lizards (*Uta stansburiana*) were captured using crappie fishing poles with a lasso finishing line at the tip, from under cover boards, or by hand. Upon capture, each lizard was examined for tick burdens on the ears and nuchal pouches. Lizards were permanently marked with a micro-brand, or temporarily marked with a small dot of nail polish on the ventral portion of the trail to ensure individuals were not sampled twice.

For Swei data:

Lizards were located by visual surveys along transect lines within the 0.5-ha sampling grid totaling 495 m at each site. Encounters included for this study were western-fence lizards (*Sceloporus occidentalis*), and southern alligator lizards (*Elgaria multicarinata*). Slip-noosing, a standard herpetological procedure, was used to sample lizards for attached ticks. All attached ticks were removed with fine-tipped forceps and placed into 70% ethanol. Each animal was individually marked allowing for abundance assessment before they were released at the point of capture. Later in lab, all recovered ticks were counted, identified to species and life stage under a stereomicroscope.

For MacDonald data:

Western fence lizards (*Sceloporus occidentalis*) are important hosts for immature *I. pacificus* (2). They are also non-competent hosts for *B. burgdorferi* s.s. in California, because they cleanse ticks of infection with the pathogen (3). *Sceloporus occidentalis* abundance was estimated using a “sight–re-sight” protocol in which five of the ten transects of each of the six trapping grids were surveyed for *S. occidentalis* between mid-March and mid-May, 2014. *Sceloporus occidentalis* were marked with a diluted latex paint mixture using a tree-marking gun (1). Three different colors of paint were used, one for each of three consecutive days to determine encounter history (1). *Sceloporus occidentalis* were also captured using nooses at each of the six sites between early March and early May 2014 in order to estimate tick burdens. Data from fire plots were omitted from analysis. Data from burned plots were excluded from this study.

For Young data:

Western fence lizard populations were surveyed using a mark-recapture approach at all plots in July of 2016 and in June of 2017 & 2018. This timing encompasses the decline from peak *I. pacificus* juvenile tick questing activity in this region that typically occurs from April through mid-June (4). At each plot, lizards were surveyed along six, fifty-meter, evenly spaced transect lines, ten-meters apart. Sighted lizards were sprayed on their dorsum with dilated latex paint mixture using an Idico hand tree-marking gun (Idico Products Co., Miami, FL, USA), enabling the marking of lizards from five to ten meters away. This marking was temporary, lasting only until the lizard's next molt (1). Surveying occurred in each plot on three consecutive days between 10 am and 4 pm in warm weather with limited wind or cloud cover, using a different paint color each day to determine a lizard’s encounter history.

In June of 2018 and 2019 any sighted lizards within the plots were captured, if possible, using a fishing pole with a fishing line-constructed noose at its end. Captured lizards were weighed, measured (snout to vent and snout to tail), and sexed. Any ticks found on the animal were removed with sterile tweezers and placed in 70% ethanol. On the lizard, the area where the tick(s) were removed was wiped down with an ethanol wipe to clean and prevent infection. In 2018 plots were searched for 60 minutes and in 2019 plots were searched for 120 minutes.

**References**

1. Swei, A., R. S. Ostfeld, R. S. Lane, and C. J. Briggs. 2011. Impact of the experimental removal of lizards on Lyme disease risk. Proceedings of the Royal Society B: Biological Sciences 278:2970–2978.
2. Casher, L., R. Lane, R. Barrett, and L. Eisen. 2002. Relative importance of lizards and mammals as hosts for Ixodid ticks in northern California. Experimental & Applied Acarology 26:127–143.
3. Lane, R. S., and G. B. Quistad. 1998. Borreliacidal factor in the blood of the western fence lizard (*Sceloporus occidentalis*). The Journal of Parasitology 84:29–34.
4. MacDonald, A. J. 2018. Abiotic and habitat drivers of tick vector abundance, diversity, phenology and human encounter risk in southern California. PLOS ONE 13:e0201665.

**Additional file 3: Supplemental Text 2. Statistical Methods justifications**

Although many climate metrics may influence our outcome of juvenile tick abundances per lizard, we want to avoid multicollinearity in our generalized additive models (GAM). To find the best fit GAM, we explored the construction of many different versions including primary climate variables (near-surface specific humidity (sph; kg/kg), maximum temperature (tmmx; °C), and minimum temperature (tmmn; °C)) or derived climate variables (drought index (pdsi)) or seasonal climate averages (mean maximum and minimum temperature in each season (spring, summer, fall, winter)) or cumulative degree days (CDD) for each year and location from January 1^st^ to March 31^st^. After many model iterations, we decided the best fit GAM that balanced biological hypotheses and simplicity included the terms: monthly maximum temperature, monthly specific humidity, and monthly drought index (pdsi). We evaluated the multicollinearity of these predictors in all models using the `car` package which produced vif < 2 (as seen in the code screen shot).


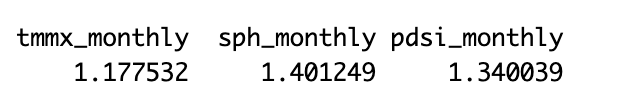


**Additional file 3: Supplemental Figure  1.** Correlation plot displaying the relationship between numeric values in the dataset. The correlation coefficient (Corr = ⍴) indicates the strength and direction of the relationship, with a perfect positive correlation of 1 shown in red and a perfect negative relationship of -1 shown in blue.
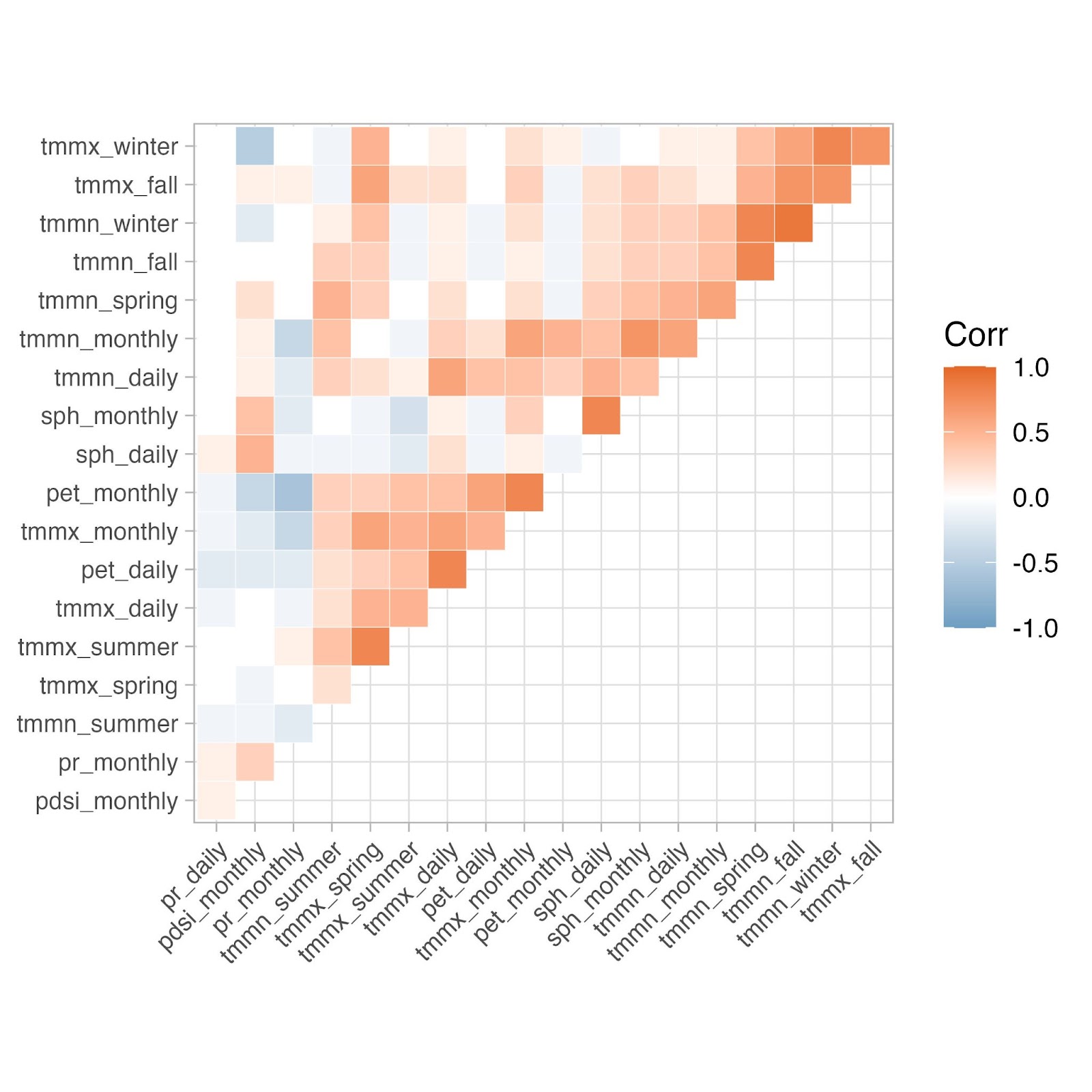

Supplement: Supplementary file 3 — Supplementary Material 3. [file 13071_2025_6749_MOESM3_ESM.docx]
