# Supplementary material for: Climate-driven variation in the phenology of juvenile Ixodes pacificus on lizard hosts
Source: Parasit Vectors. 2025 Apr 15;18:141. doi: 10.1186/s13071-025-06749-4 (PMC12001419; doi:10.1186/s13071-025-06749-4)
Supplement: Supplementary file 4 — Supplementary Material 4. [file 13071_2025_6749_MOESM4_ESM.docx]

**Climate-driven variation in the phenology of juvenile *Ixodes pacificus* on lizard hosts**

**Samantha Sambado^1*^, Amanda Sparkman^2^, Andrea Swei^3^, Andrew J MacDonald^4^, Hillary S Young^1^, Jordan Salomon^5^, Arielle Crews^6^, Kacie Ring^1^, Stephanie Copeland^1^, and Cheryl J Briggs^1^**

1. Ecology, Evolution & Marine Biology Department at University of California Santa Barbara, Santa Barbara, California, USA

2. Biology Department at Westmont College, Santa Barbara, California, USA

3. Biology Department at San Francisco State University, California, USA

4. Bren School of Environmental Science & Management at University of California Santa Barbara, California, USA

5. Ecology & Evolutionary Biology Program at Texas A&M University, College Station, Texas, USA

6. San Mateo County Mosquito and Vector Control, Burlingame, California, USA

***Correspondence**: [sbsambado@ucsb.edu](mailto:sbsambado@ucsb.edu)

**Supplementary information**

TABLE OF CONTENTS

**Additional file 1: Sampling locations**

**Table S1.** Location coordinates

**Table S2.** Location sample dates

**Additional file 2: Location characteristics**

**Figure S1.** Location sampling frequency

**Figure S2.** Lizards

**Table S1.** mean and sd per location

**Additional file 3: Method details**

**Text S1.** Additional details on Field methods

**Text S2.** Statistical method justifications

**Figure S1.** Covariate correlation and vif results

**Additional file 4: Phenological metrics by climate regions**

**Figure S1.** Distribution of juvenile burdens by CR3

**Figure S2.** Ticks per month and year by CR5

**Table S1.** Phenology metrics for all climate regions

**Additional file 5: GAM results and diagnostics**

**Figure S1.** GAM 1 results and diagnostics

**Figure S2.** GAM 2 results and diagnostics

**ADDITIONAL FILE 4: Phenological metrics by climate regions**

**Additional file 4: Figure S1.** Histogram showing the distribution of mean abundances of (A) larvae and (B) nymphs on individual lizards by climate regions.

**
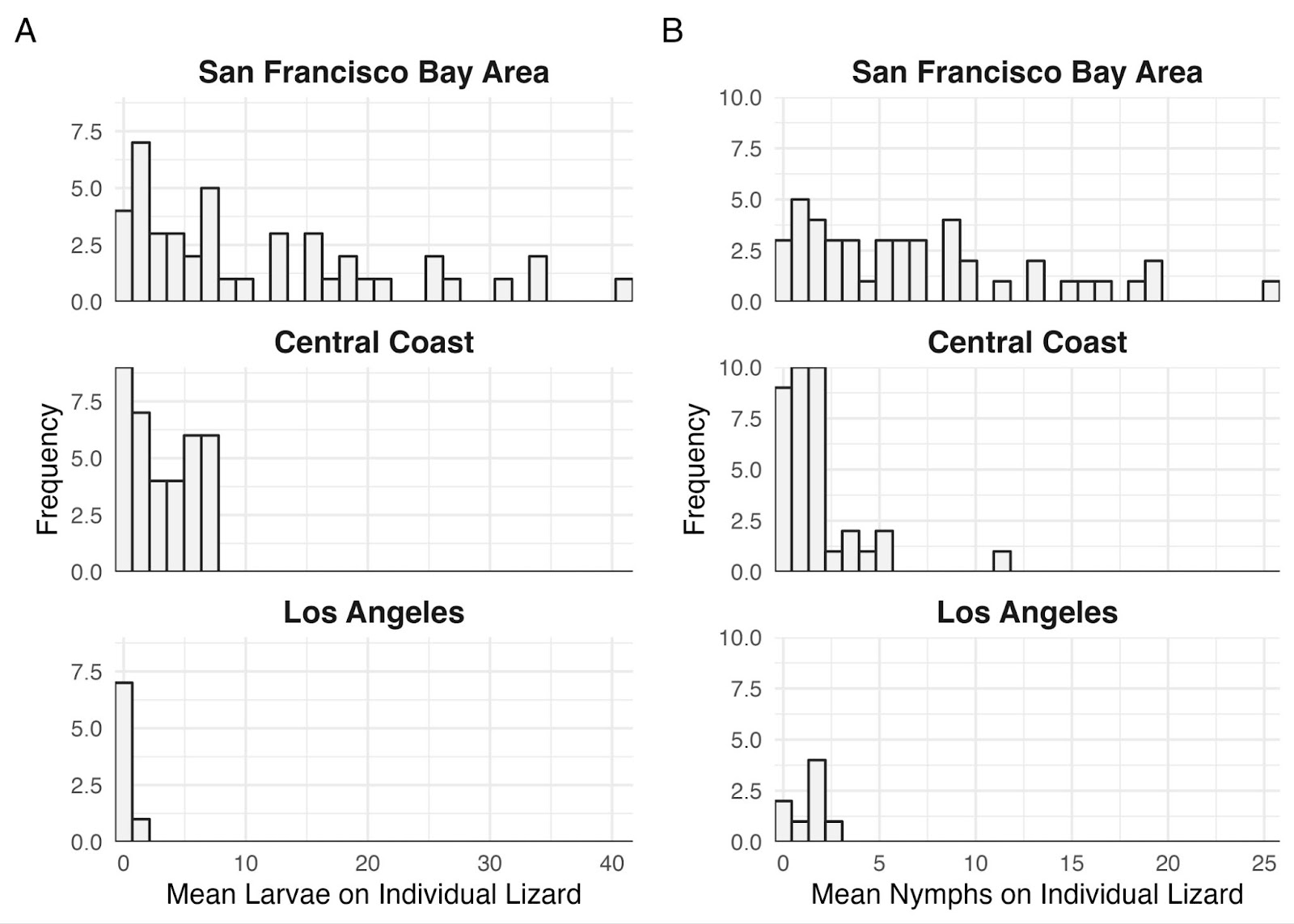
**

**Additional file 4: Figure S2.** Box plots illustrating the abundance of juvenile ticks on individual lizards, by month and year. Points are colored-coded by climatic region.


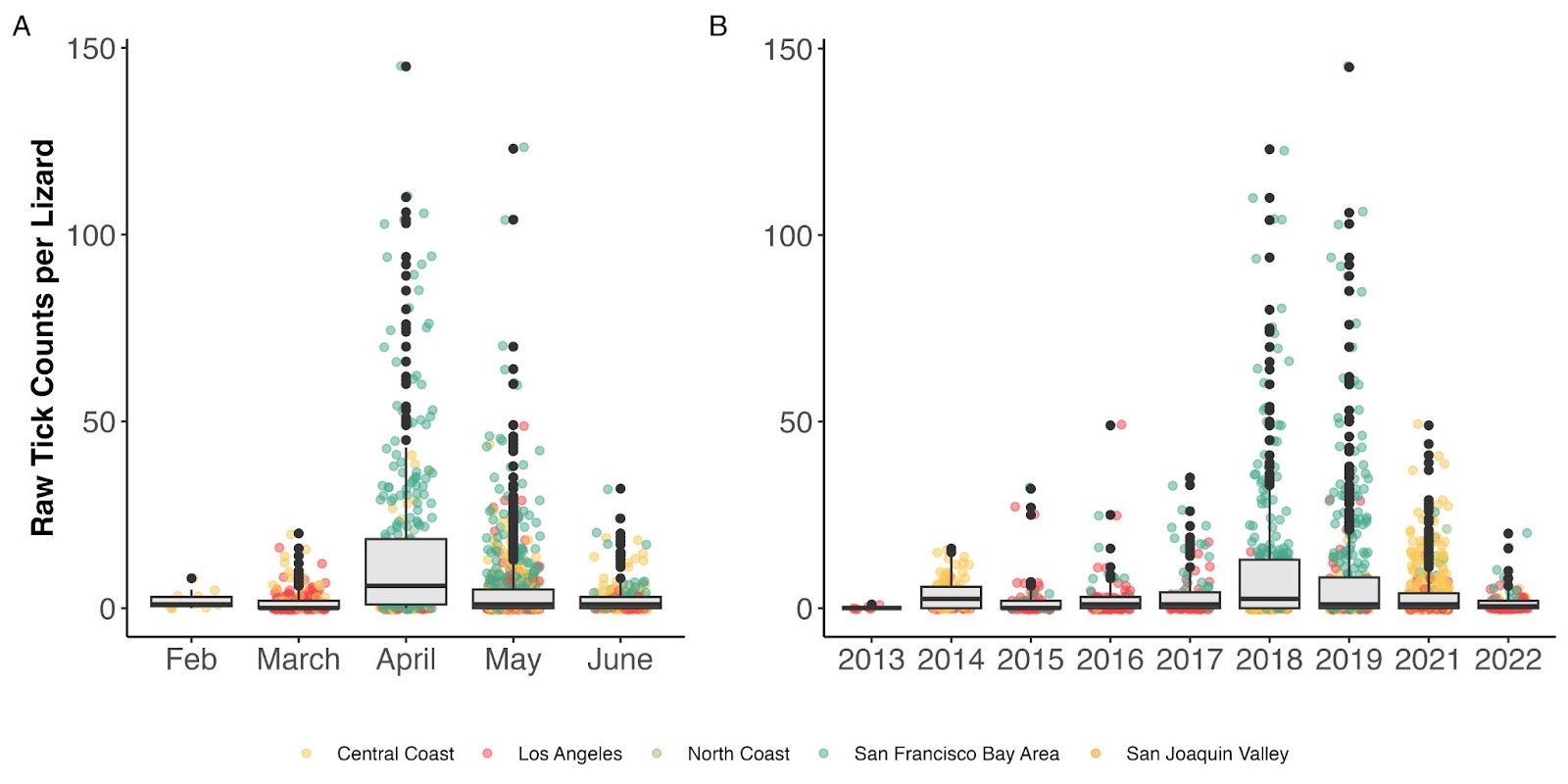


**Additional file 4: Table S1.** Phenological metrics for larvae, nymphs, and total ticks (larvae and nymphs) for all climate regions. KDE = kernel density estimation.  Climate regions (CR) are: NC = North Coast, SF = San Francisco Bay Area, CC = Central Coast, SJV = San Joaquin Valley, LA = Los Angeles.

|  |  | **NC** | **SF** | **SJV** | **CC** | **LA** |
| --- | --- | --- | --- | --- | --- | --- |
| **Abundance**  (mean± sd) | Larvae | 2.58 ± 3.58 | 12.6 ± 19.1 | NA | 3.32 ± 5.5 | 0.32 ± 0.9 |
|  | Nymphs | 4.25 ± 5.61 | 9.39 ± 10.7 | NA | 2.56 ± 3.9 | 1.25 ± 1.7 |
|  | Total | 6.83 ± 6.39 | 18.40 ± 23.07 | 0.92 ± 1.85 | 3.84 ± 6.62 | 1.64 ± 4.0 |
| **Overlap Metrics** | KDE | 0.42 | 0.89 | NA | 0.32 | 0.30 |
|  | Jaccard Index | 0.55 | 0.47 | NA | 0.38 | 0.22 |
| **Peak Mean Burden**  (Julian date) | Larvae | 97 | 144 | NA | 101 | 104 |
|  | Nymphs | 97 | 119 | NA | 101 | 104 |
|  | Total | 97 | 110 | 110 | 101 | 131 |
| **Phenological Dates**  (Julian date) | *Start date* | | | | | |
|  | Larvae | 97 | 94 | NA | 63 | 83 |
|  | Nymphs | 97 | 94 | NA | 63 | 83 |
|  | Total | 97 | 94 | 110 | 63 | 63 |
|  | *End date* | | | | | |
|  | Larvae | 125 | 156 | NA | 160 | 164 |
|  | Nymphs | 153 | 156 | NA | 160 | 164 |
|  | Total | 153 | 173 | 135 | 174 | 176 |
|  | *Length of season* | | | | | |
|  | Larvae | 28 | 62 | NA | 97 | 81 |
|  | Nymphs | 56 | 62 | NA | 97 | 81 |
|  | Total | 56 | 79 | 25 | 111 | 113 |
