# Supplementary material for: Climate-driven variation in the phenology of juvenile Ixodes pacificus on lizard hosts
Source: Parasit Vectors. 2025 Apr 15;18:141. doi: 10.1186/s13071-025-06749-4 (PMC12001419; doi:10.1186/s13071-025-06749-4)

**Climate-driven variation in the phenology of juvenile *Ixodes pacificus* on lizard hosts**

**Samantha Sambado^1*^, Amanda Sparkman^2^, Andrea Swei^3^, Andrew J MacDonald^4^, Hillary S Young^1^, Jordan Salomon^5^, Arielle Crews^6^, Kacie Ring^1^, Stephanie Copeland^1^, and Cheryl J Briggs^1^**

1. Ecology, Evolution & Marine Biology Department at University of California Santa Barbara, Santa Barbara, California, USA

2. Biology Department at Westmont College, Santa Barbara, California, USA

3. Biology Department at San Francisco State University, California, USA

4. Bren School of Environmental Science & Management at University of California Santa Barbara, California, USA

5. Ecology & Evolutionary Biology Program at Texas A&M University, College Station, Texas, USA

6. San Mateo County Mosquito and Vector Control, Burlingame, California, USA

***Correspondence**: [sbsambado@ucsb.edu](mailto:sbsambado@ucsb.edu)

**Supplementary information**

TABLE OF CONTENTS

**Additional file 1: Sampling locations**

**Table S1.** Location coordinates

**Table S2.** Location sample dates

**Additional file 2: Location characteristics**

**Figure S1.** Location sampling frequency

**Figure S2.** Lizards

**Table S1.** mean and sd per location

**Additional file 3: Method details**

**Text S1.** Additional details on Field methods

**Text S2.** Statistical method justifications

**Figure S1.** Covariate correlation and vif results

**Additional file 4: Phenological metrics by climate regions**

**Figure S1.** Distribution of juvenile burdens by CR3

**Figure S2.** Ticks per month and year by CR5

**Table S1.** Phenology metrics for all climate regions

**Additional file 5: GAM results and diagnostics**

**Figure S1.** GAM 1 results and diagnostics

**Figure S2.** GAM 2 results and diagnostics

**ADDITIONAL FILE 5: GAM model results and diagnostics**

**Additional file 5: Figure S1.** GAM model results and diagnostics for larval abundance per lizard. The autocorrelation smoothing spline s(lat, lon) was unable to converge so this model does not correct for autocorrelation, but still includes the smooth terms for primary climate variables.


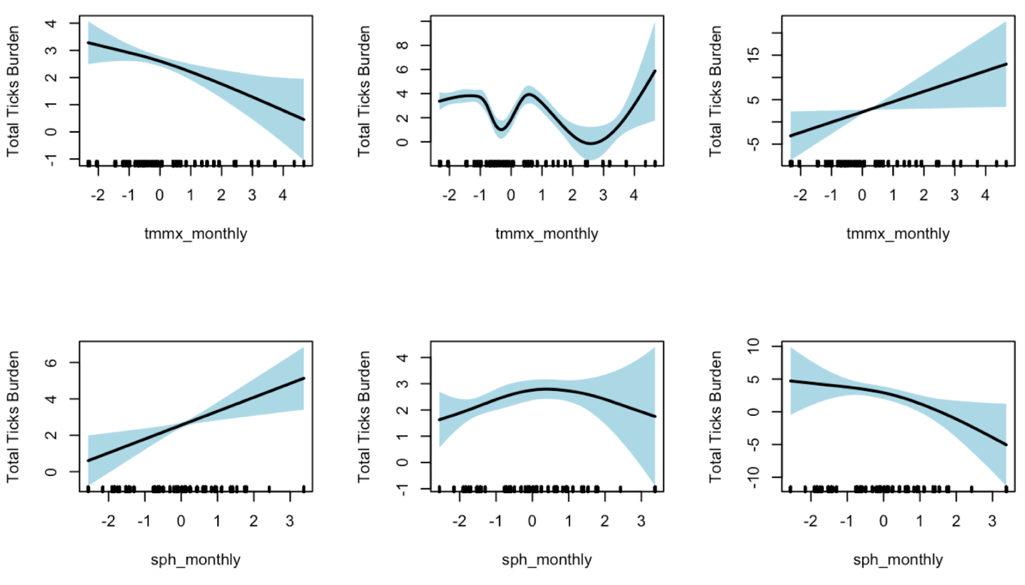

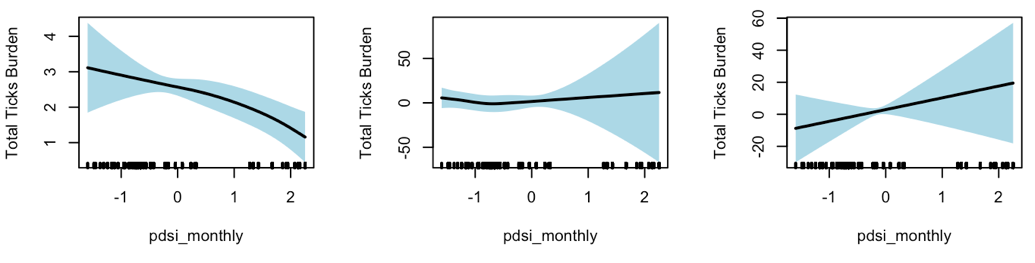


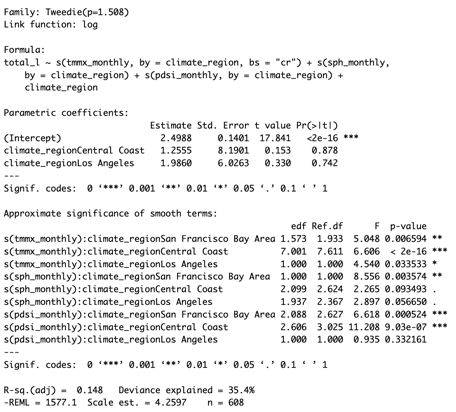


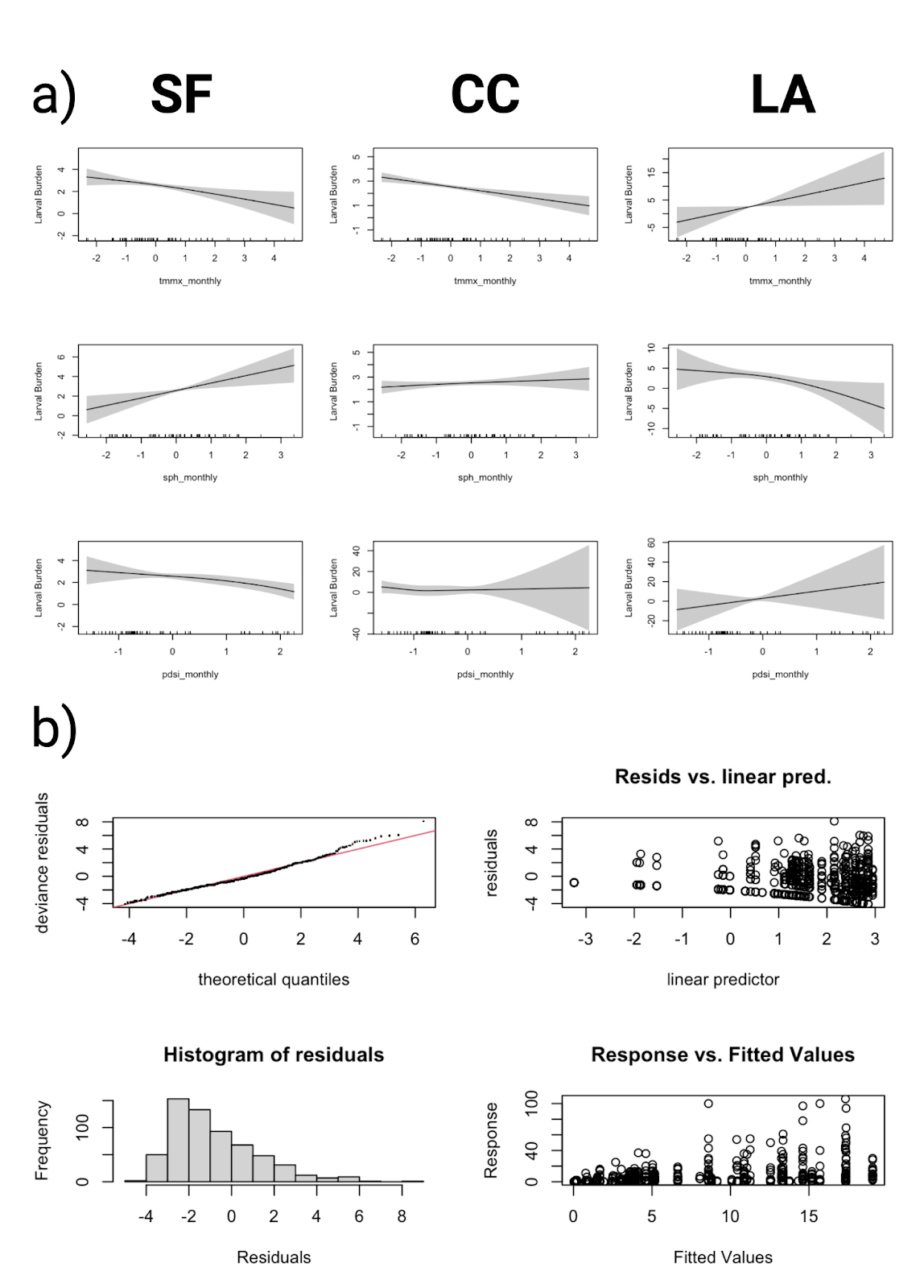


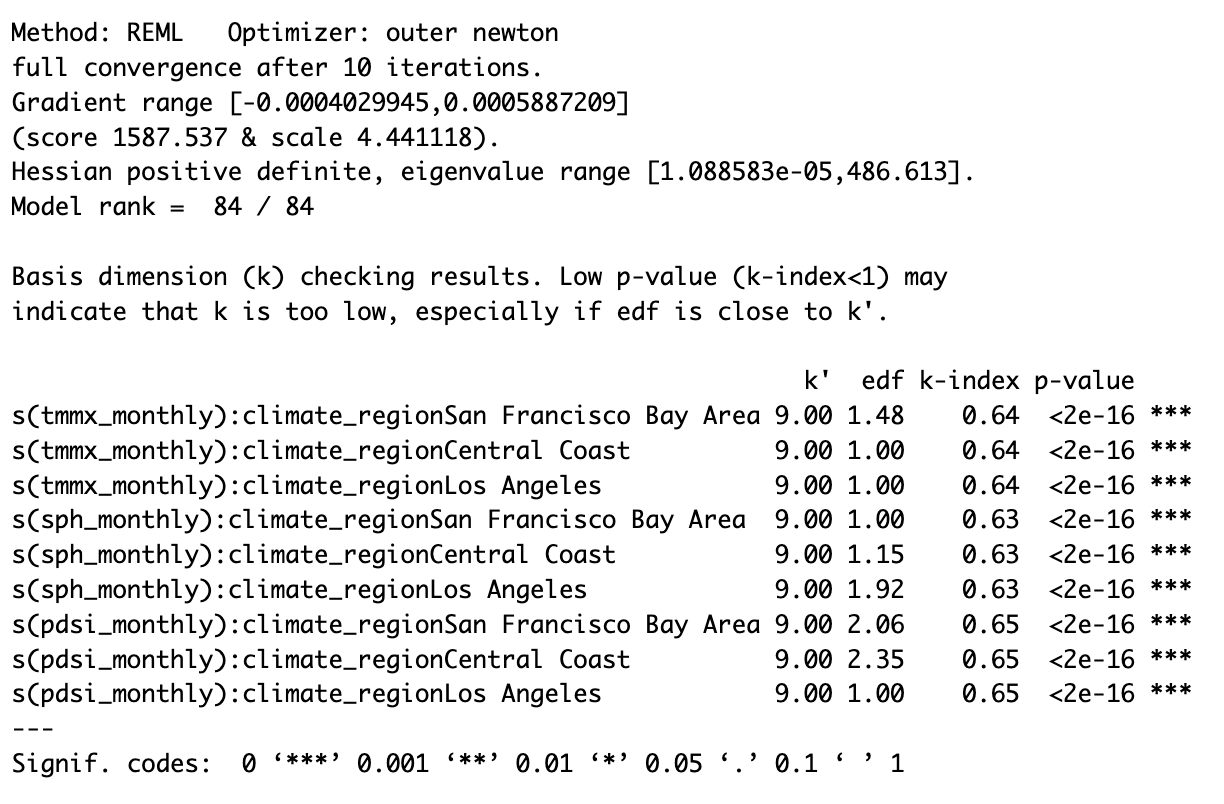


**Additional file 5: Figure S2.** GAM model results and diagnostics for nymphal abundance per lizard. The autocorrelation smoothing spline s(lat, lon) was unable to converge so this model does not correct for autocorrelation, but still includes the smooth terms for primary climate variables.


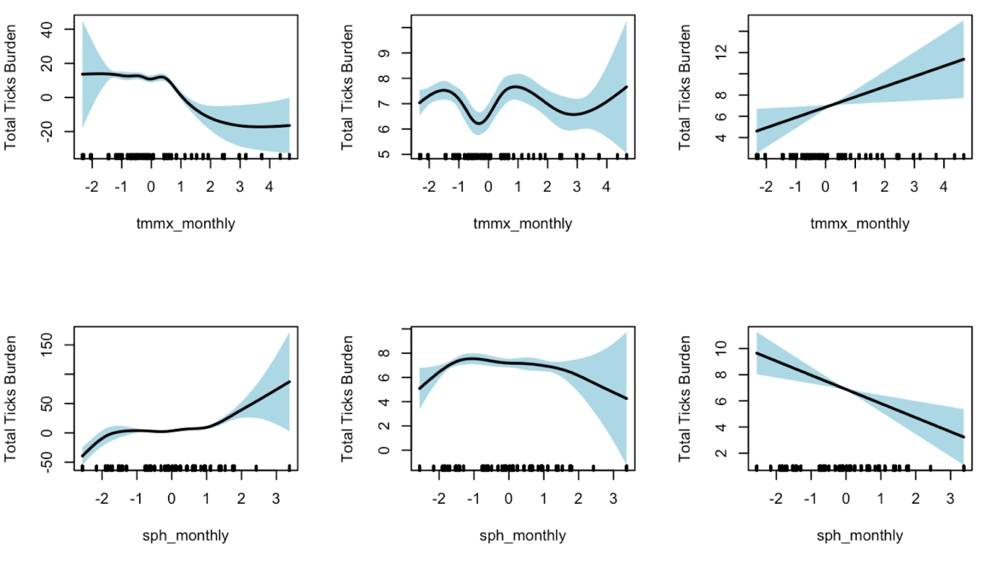

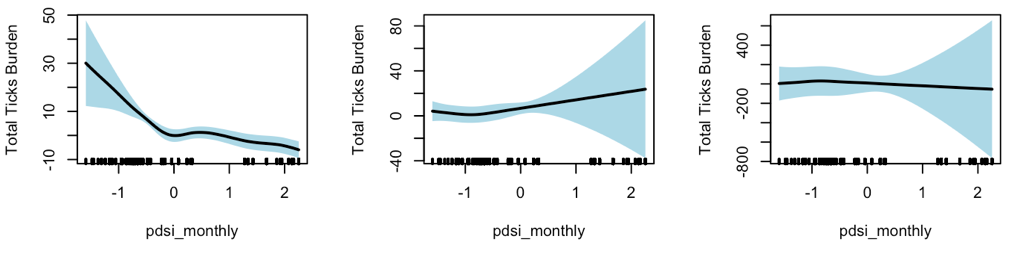


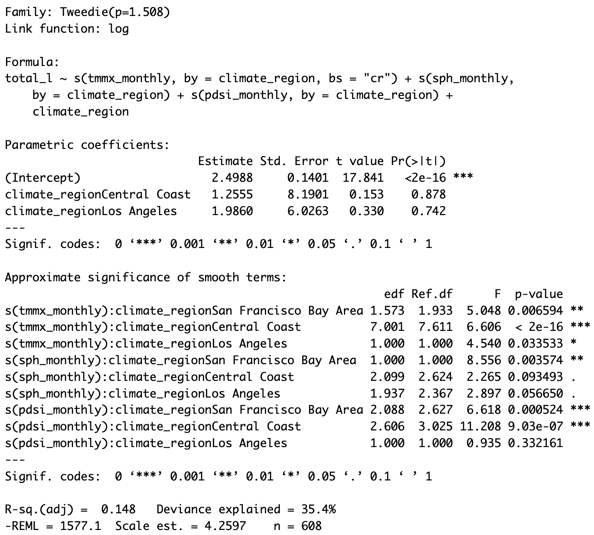


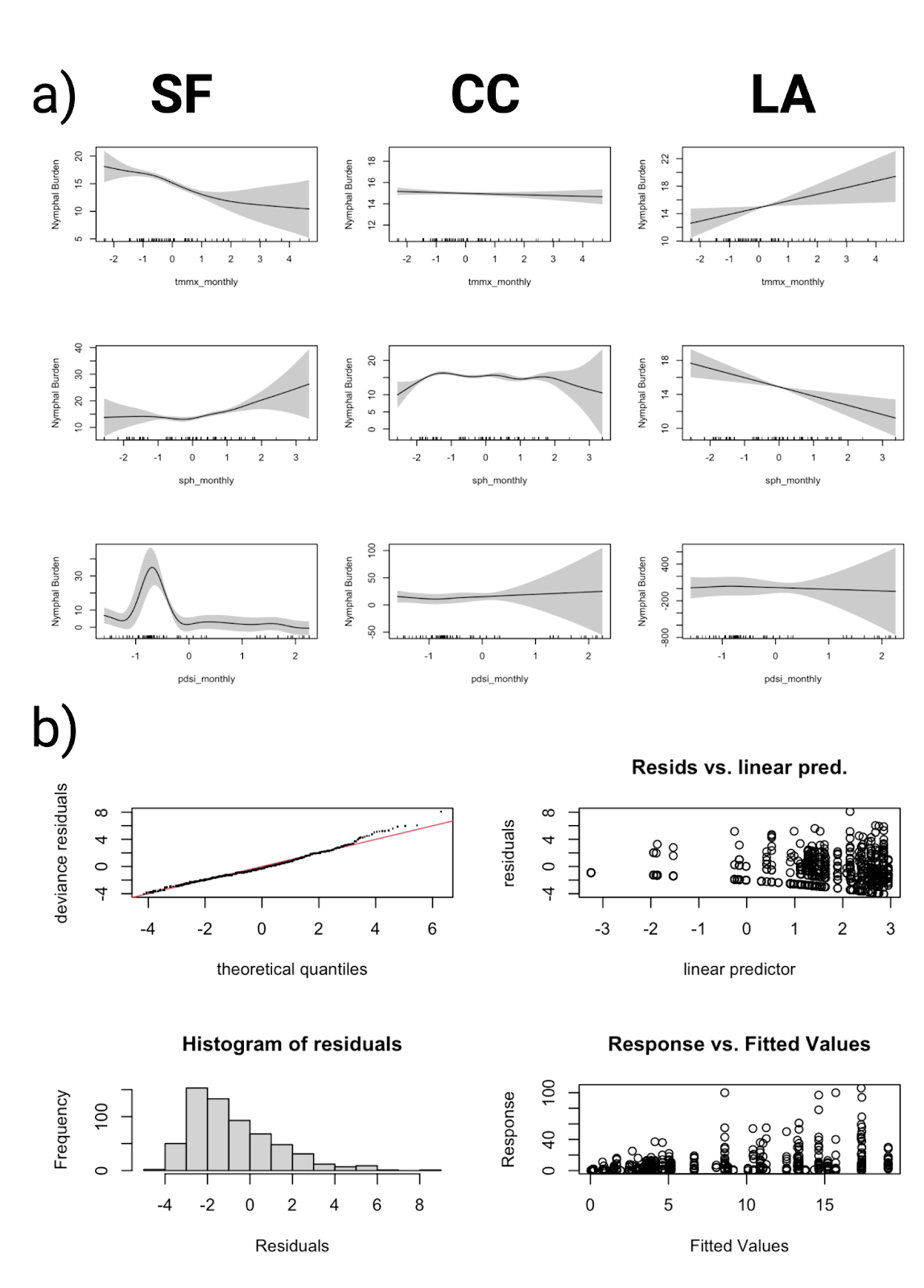


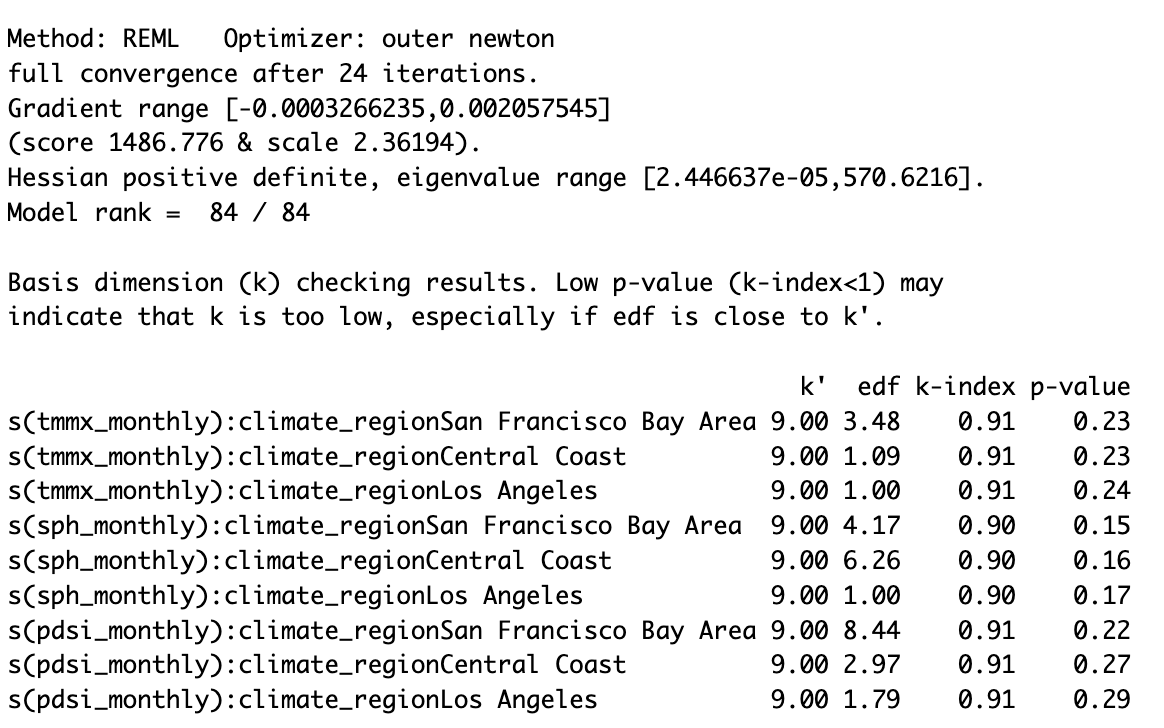

Supplement: Supplementary file 5 — Supplementary Material 5. [file 13071_2025_6749_MOESM5_ESM.docx]
